# Optogenetic Control of PIP_3_: PIP_3_ Is Sufficient to Induce the Actin-Based Active Part of Growth Cones and Is Regulated via Endocytosis

**DOI:** 10.1371/journal.pone.0070861

**Published:** 2013-08-07

**Authors:** Toshiyuki Kakumoto, Takao Nakata

**Affiliations:** 1 Department of Cell Biology, Tokyo Medical and Dental University, Tokyo, Japan; 2 The Center for Brain Integration Research (CBIR), Tokyo Medical and Dental University, Tokyo, Japan; Temple University, United States of America

## Abstract

Phosphatidylinositol-3,4,5-trisphosphate (PIP_3_) is highly regulated in a spatiotemporal manner and plays multiple roles in individual cells. However, the local dynamics and primary functions of PIP_3_ in developing neurons remain unclear because of a lack of techniques for manipulating PIP_3_ spatiotemporally. We addressed this issue by combining optogenetic control and observation of endogenous PIP_3_ signaling. Endogenous PIP_3_ was abundant in actin-rich structures such as growth cones and “waves”, and PIP_3_-rich plasma membranes moved actively within growth cones. To study the role of PIP_3_ in developing neurons, we developed a PI3K photoswitch that can induce production of PIP_3_ at specific locations upon blue light exposure. We succeeded in producing PIP_3_ locally in mouse hippocampal neurons. Local PIP_3_ elevation at neurite tips did not induce neurite elongation, but it was sufficient to induce the formation of filopodia and lamellipodia. Interestingly, ectopic PIP_3_ elevation alone activated membranes to form actin-based structures whose behavior was similar to that of growth-cone-like “waves”. We also found that endocytosis regulates effective PIP_3_ concentration at plasma membranes. These results revealed the local dynamics and primary functions of PIP_3_, providing fundamental information about PIP_3_ signaling in neurons.

## Introduction

Phosphatidylinositol-3,4,5-trisphosphate (PIP_3_) is an intracellular signaling lipid with multiple roles in various cellular functions [1], [2]. It is produced from phosphatidylinositol bisphosphate (PI(4,5)P_2_) by phosphatidylinositol 3-kinases (PI3Ks) on the plasma membrane, and rapidly dephosphorylated by the tumor suppressor phosphatase and tensin homolog (PTEN) [1]. PIP_3_ comprises only a fraction of all phospholipids in the resting state [2]. The distribution of PIP_3_ is thought to be deeply related to its functions [3], [4]. In neurons, PIP_3_ has been implicated in growth cone guidance [5], [6], the formation of filopodia and branches in axons [7], and the formation of axons [8], [9]. Considering the rapid and strict control of PIP_3_ levels [2], it is necessary to control PIP_3_ production both spatially and temporally. However, methods used in previous studies, such as pharmacological inhibition [8], [9], neurotrophic factors [6], [7], and genetic perturbation [10], have limited ability to provide specificity of the location and timing of effects.

Among approaches to overcome these limitations, recently developed optogenetic tools are promising. They allow us to rapidly control the amplitude, location and timing of activity of specific signals rapidly [11]. They also diminish the problem of variance among cells, because we can assess the effects of activated signaling on the same cells. Accordingly, using these tools enables us to obtain well controlled data on the effects of acute activation of signaling pathways in cells. Such data, together with knowledge of the cellular distribution of signals, will reveal the spatiotemporal role of these signals in cells.

We first observed the endogenous distribution and dynamics of PIP_3_ in developing mouse hippocampal neurons. PIP_3_ was abundant at growth cones and growth cone-like “waves” [12], and its level seemed to be related to growth cone size and dynamics. PIP_3_-rich membranes moved dynamically within growth cones.

To induce acute and localized PIP_3_ signaling, we developed a PI3K photoswitch that enables PIP_3_ to be produced at specific locations upon blue light exposure. We succeeded in producing PIP_3_ locally in neurons. Local accumulation of PIP_3_ induced the formation of dynamic F-actin-related structures and enlarged growth cones, but did not cause neurites to be elongated. Interestingly, ectopic production of PIP_3_ induced growth-cone-like structures in the middle of neurites and soma. We also found that PIP_3_-rich membranes were endocytosed.

## Results

### Construction and Characterization of the PI3K Photoswitch

**Figure 1:**
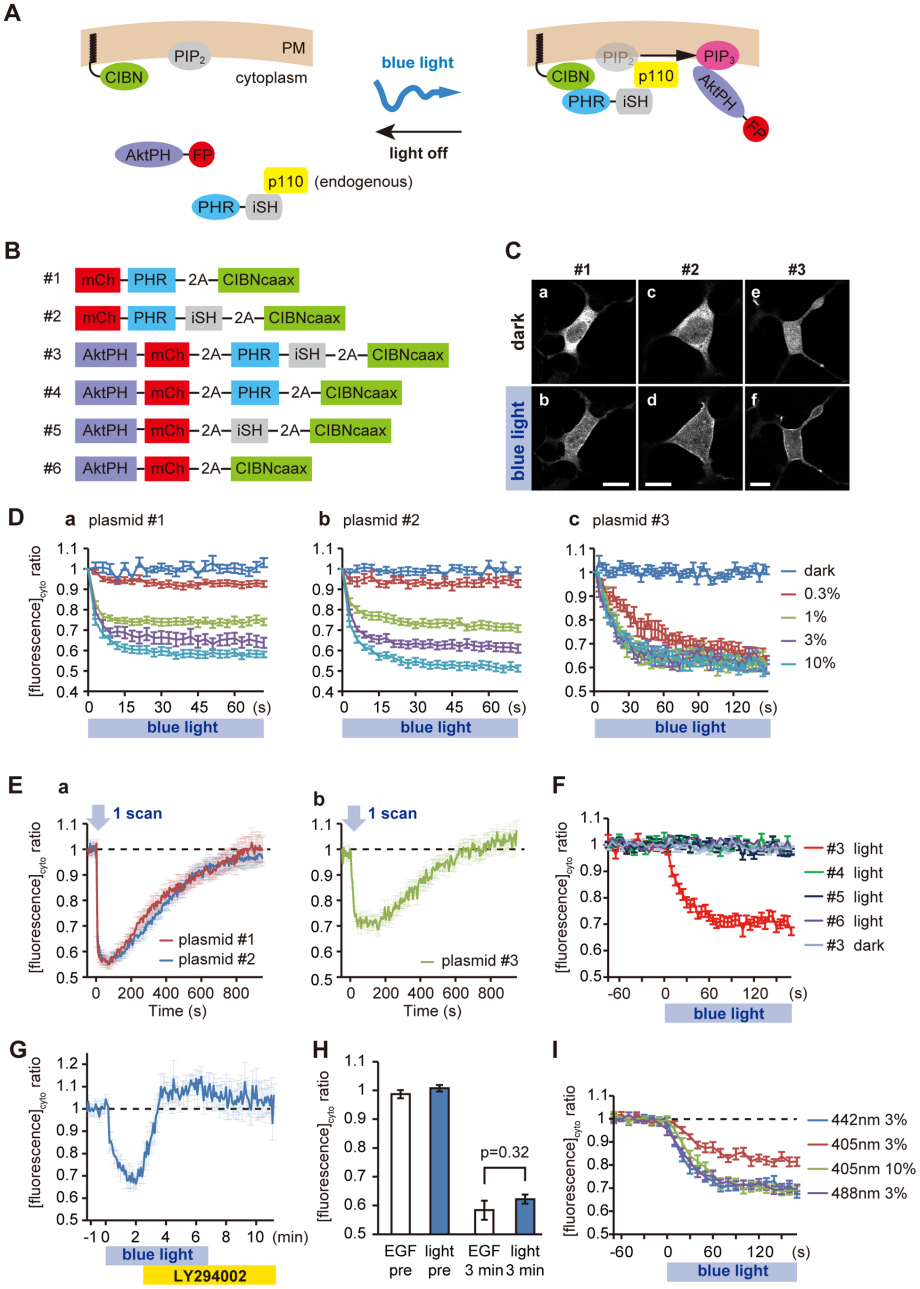
Design and characterization of the PI3K photoswitch. (A,B) Schematic design of the PI3K photoswitch and constructs used. (C) Fluorescence images of HEK293 cells expressing plasmid #1, #2 or #3 before and after photoactivation (3% of 442-nm HeCd laser). Scale bars, 10 µm. (D–I) Membrane translocation assay based on the change in cytoplasmic fluorescence. (D) Time-course of changes in cytoplasmic mCh-PHR (a), mCh-PHR-iSH (b) and AktPH-mCh (c) fluorescence under different intensities of light (442 nm, 0.33 Hz). Note that PIP_3_ production became saturated under weak photoactivation (such as 1% of a HeCd laser). (E) Time-course of changes in cytoplasmic mCh-PHR (red line in a), mCh-PHR-iSH (blue line in a) and AktPH-mCh (b) fluorescence after one pulse of 442-nm laser (3%). (F) Control study using domain-deleted controls (plasmid #4–6 light) or under no photoactivation (plasmid #3 dark). Membrane recruitment of AktPH-mCh was iSH (#4, 6), PHR (#5, 6), and blue light dependent. (G) Time-course of changes in cytoplasmic AktPH-mCh fluorescence in the course of photoactivation (3% of 442-nm laser, 0.2 Hz) followed by application of LY294002 (50 µM). (H) Comparison of PIP_3_ production in HEK293 cells between EGF application (50 ng/ml) and photoactivation of the PI3K photoswitch (3% of 442-nm laser, 0.2 Hz). (I) Comparison of PIP_3_ production under different laser wavelengths and powers. The graph indicated by “442 nm 3%” used the same data as the graph shown in (F) indicated by “#3 light”. Data are expressed as means ± s.e.m. in all panels. See **Movie S1**.

It was recently reported that the photolyase homology region (PHR) of cryptochrome 2 (CRY2) and CIBN (truncated cryptochrome-interacting basic-helix-loop-helix 1) require no exogenous chromophores and dimerize within seconds upon blue light exposure in mammalian cells [13]. We developed a PI3K photoswitch based on a PI3K chemical activation system [14] and the PHR-CIBN module. The overall design of the PI3K photoswitch is as follows (**Fig. 1A**). CIBN is targeted to the plasma membrane by a C-terminally fused K-ras CAAX motif (CIBNcaax). Before photoactivation, PHR-iSH (containing the inter-SH2 domain of p85β, the regulatory subunit of PI3K) exists in the cytoplasm where it interacts with endogenous p110, the catalytic subunit of PI3K. Upon blue light exposure, the PHR-iSH–p110 complex moves rapidly to the plasma membrane, triggering PIP_3_ production. The generation of PIP_3_ is indicated by the translocation of mCherry-tagged AktPH, the pleckstrin homology domain of Akt, which specifically binds to PIP_3_ [15]. We used a 2A peptide sequence to co-express multiple, discrete proteins in a single ORF [16] (**Fig. 1B**). Although similar optogenetic systems for controlling PI3K production have been reported [17], [18], our system using the 2A peptide sequence has at least two advantages: (1) a single fluorescent tag is sufficient for checking the expression of the switch; and (2) equimolar expression of the two components ensures more robust reproducibility than if the two plasmids were co-transfected.

We first characterized light-induced translocation of PHR and PHR-iSH to the membranes in HEK293 cells. Both PHR (**Fig. 1B** **plasmid #1**) and PHR-iSH (**Fig. 1B** **plasmid #2**) tagged with mCherry (mCh) showed cytoplasmic localization before photoactivation (**Fig. 1C** **a,c**). Blue light irradiation triggered rapid membrane translocation of mCh-PHR (t_1/2_ = 7.6±1.8 s, n = 25) or mCh-PHR-iSH (t_1/2_ = 6.8±2.0 s, n = 8, **Fig. 1C** **b,d**), indicating that the insertion of 2A peptide sequences did not affect the localization or light-induced heterodimerization of the peptides used. We estimated the amount of membrane translocation of the fluorescent protein by measuring the decrease in fluorescence in the cytoplasmic fraction [13]. The difference in t_1/2_ between mCh-PHR and mCh-PHR-iSH was not statistically significant (p = 0.38, two-sample t-test). mCh-PHR-iSH and mCh-PHR bound to CIBNcaax in proportion to the light intensity (**Fig. 1D** **a,b**). The recovery half times for intracellular mCh fluorescence were 295 s and 324 s for PHR (n = 13) and PHR-iSH (n = 18), respectively (**Fig. 1E** **a**).

Next, we co-expressed the PI3K photoswitch and AktPH-mCh (**Fig. 1B** **plasmid #3**) in HEK293 cells to test whether this system could work. Before photoactivation, AktPH-mCh showed a cytoplasmic localization, which suggested that there was little leakage of PI3K activity in the dark state (**Fig. 1C** **e**). Upon blue light exposure (442-nm HeCd laser, 3%, 0.2 Hz), AktPH-mCh rapidly (t_1/2_ = 25.5±6.6 s, n = 18) moved to the plasma membrane (**Fig. 1C** **f**). This membrane translocation was strictly dependent on the PI3K photoswitch based on the following observations: (1) it required irradiation (**Fig. 1F** **#3 dark**); (2) it required both iSH and PHR (**Fig. 1F** **#4, 5, 6 light**); (3) it was blocked by application of PI3K inhibitor (LY294002) (**Fig. 1G**); and (4) the amount of PIP_3_ production was correlated with the intensity of light (**Fig. 1D** **c**). It took about 10 minutes for the PIP_3_ signal to recover to the pre-photoactivation level (**Fig. 1E** **b**, n = 18). This seemed to be largely due to the time-course of PHR-CIBN dissociation, because the level of cytoplasmic AktPH-mCh increased in accordance with the level of cytoplasmic mCh-PHR-iSH (**Fig. 1E** **a**). The amount of PIP_3_ produced in the presence of a saturated level of PI3K photoswitch photoactivation was comparable to that following EGF application, indicating that the photoswitch can activate endogenous PI3K to the physiologically appropriate degree (**Fig. 1H**). We also tested whether other wavelengths of light could photoactivate the photoswitch (**Fig. 1I**). Both 3% of a 488-nm Argon laser and 10% of a 405-nm diode laser (0.1 Hz) could activate the photoswitch to its saturated level (442 nm 3%, 0.2 Hz). We succeeded in repetitively inducing PIP_3_ production using this system (**Movie S1**).

Collectively, these findings suggest that our photoswitch had little leakage in the dark state, and could be used to produce PIP_3_ in a strictly light-dependent manner to the level of physiological stimulation.

### Distribution and Dynamics of Endogenous PIP_3_ in Mouse Hippocampal Neurons

We then examined the distribution and dynamics of endogenous PIP_3_ in mouse hippocampal neurons. In cultures of rodent embryonic hippocampal neurons, several hours after plating, isolated neurons typically bear several highly dynamic neurites tipped by mobile growth cones that have lamellipodia and filopodia (stage 2). Then, without the addition of any exogenous factors, one of the neurites rapidly grows and becomes an axon, while the other neurites remain short (stage 3) [19].

**Figure 2:**
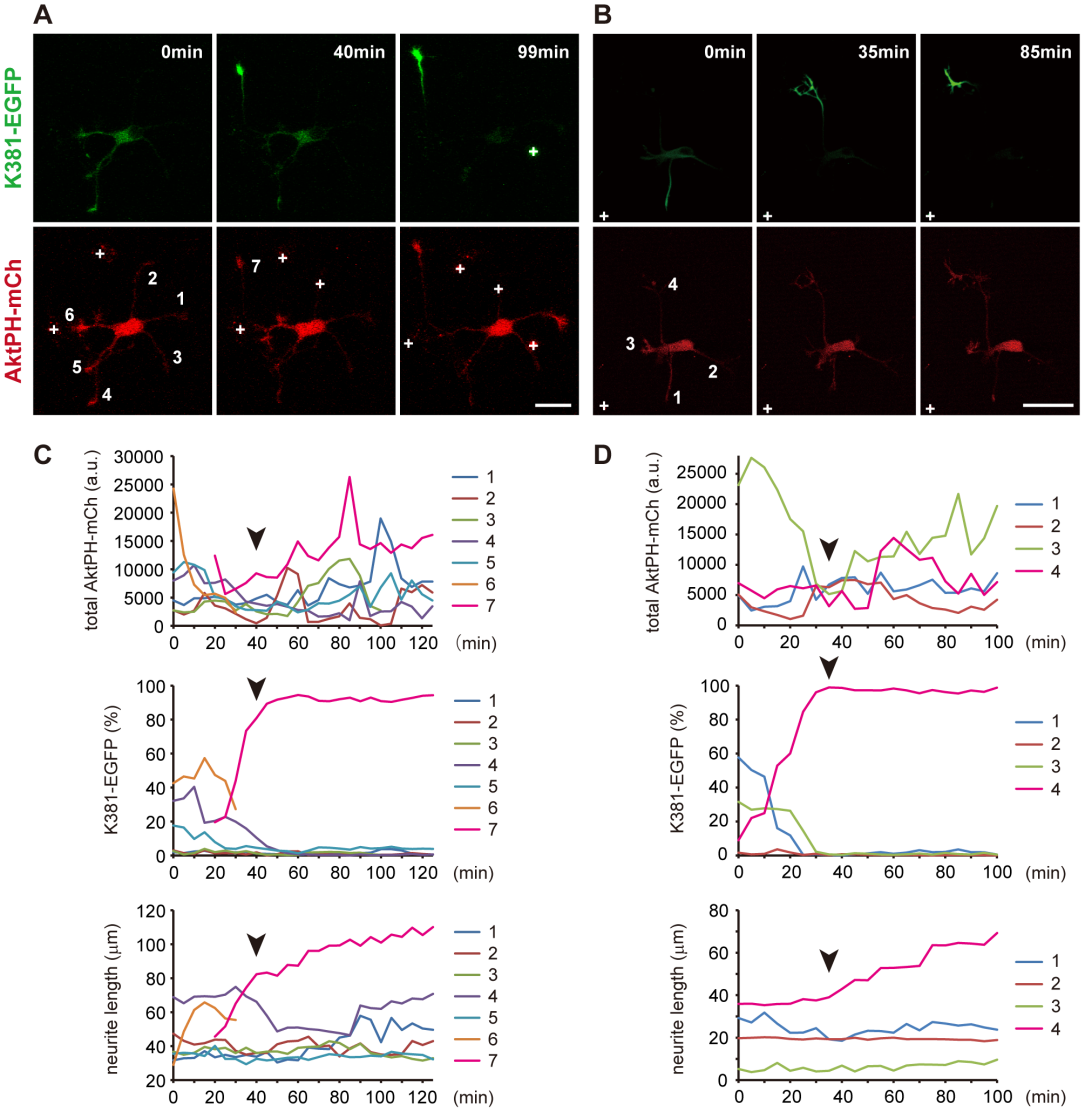
Dynamics of PIP_3_ signaling during axon formation. (A,B) Two representative images of hippocampal neurons expressing AktPH-mCh (red) and K381-EGFP (green), an early axonal marker. See **Movies S2, S3**. (C,D) Time-course of changes in the levels of AktPH-mCh (upper graphs) and K381-EGFP (middle graphs) in each growth cone, and in neurite length (lower graphs) during the formation of axons. The K381-EGFP level in each growth cone is shown as a percentage of the total for all growth cones [21]. The graphs in (C) and (D) correspond to the neurons in (A) and (B), respectively. The same color in the graphs indicates the same neurites numbered in (A) and (B). Note that some data for the graphs in (C) could not be obtained. Data on neurite 7 before 20 min was not recorded, because it branched from neurite 6 at that time. The AktPH-mCh level in neurite 6 was also not recorded after 35 min, because it barely had a growth cone. The AktPH-mCh and K381-EGFP levels in neurite 3 were not recorded after 100 min because of a bright particle attached to the growth cone. Arrowheads indicate the exact time of the formation of axons. Scale bars represent 30 µm. White crosses in A and B mark cell debris.

We investigated the dynamics of endogenous PIP_3_ during axon formation at the whole cell level by co-expressing AktPH-mCh and the early axonal marker K381-EGFP (truncated conventional kinesin) [20]–[22] in neurons. An axon was deemed to have formed when one neurite rapidly extended and became >15 µm longer than any other neurites (**Fig. 2A–D**) [21]. K381 accumulated at the tips of future axons before neurite elongation (**Fig. 2A,B** **and Movies S2, S3**). AktPH-mCh was rich in large, motile growth cones as reported previously [9], rather than in the future axons (**Fig. 2A,B** **and Movies S2, S3**). The amount of PIP_3_ at the tips of the neurites changed on a timescale of tens of minutes (**Fig. 2B,D**). During axon formation, the level of PIP_3_ was not related to the fate of a neurite: in one neuron it was highest in the future axon (**Fig. 2A,C**), while in another neuron the level was higher in neurites not fated to become an axon than in the future axon (**Fig. 2B,D**). Even in the former case, PIP_3_ production was not inhibited in the neurites that did not eventually become an axon (**Fig. 2C** **, Movie S2**, most notably seen in neurite 1). These observations question the notion that local positive feedback of PIP_3_ in one neurite induces axon formation, and that global negative regulation of PIP_3_ in the other neurites suppresses them from becoming axons [**23**].

**Figure 3:**
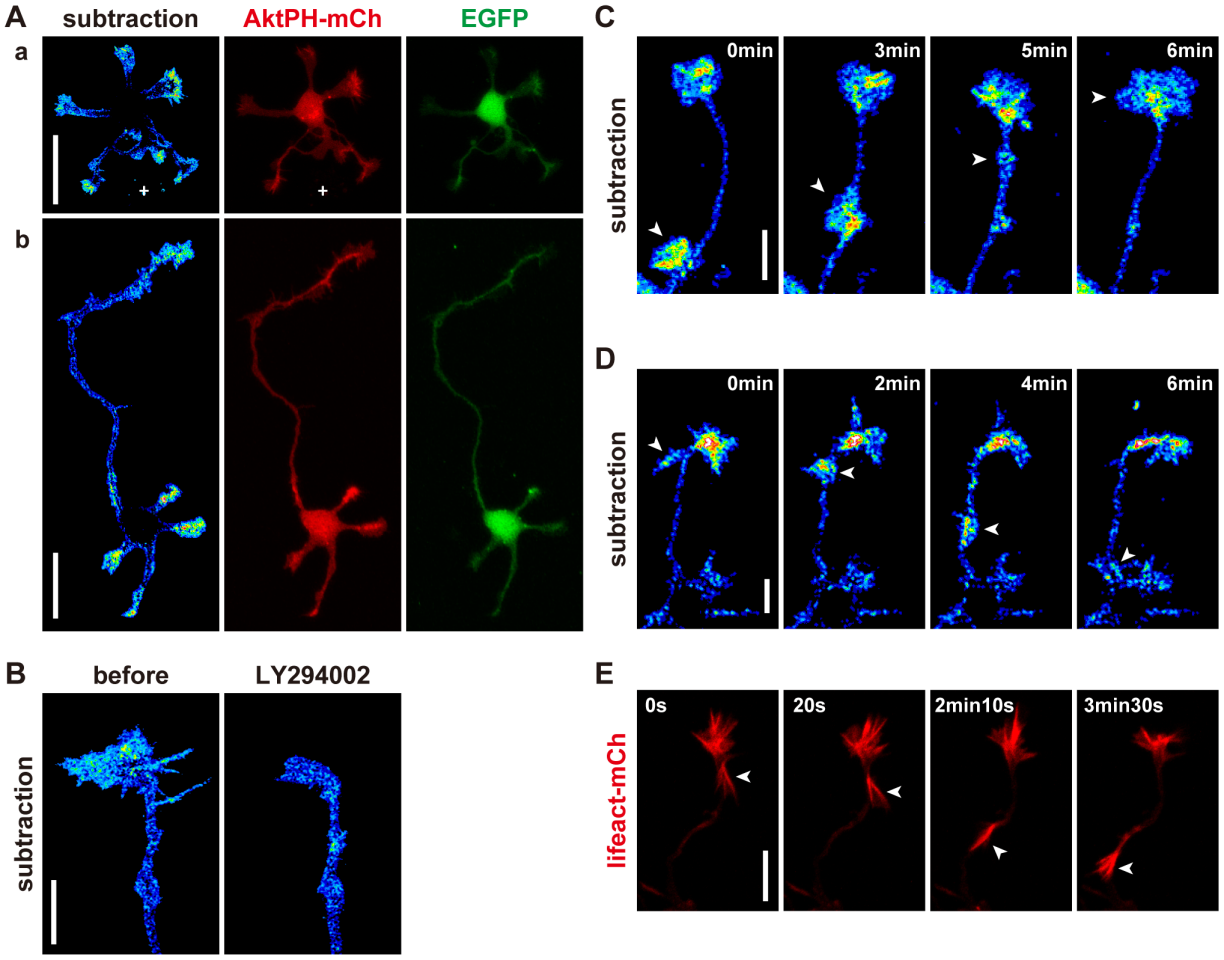
Distribution of endogenous PIP_3_ in developing neurons. (A) Subtracted AktPH (pseudo-color), AktPH-mCh (red) and EGFP (green) images of hippocampal neurons at stage 2 (a) and early stage 3 (b). White crosses in (a) mark cell debris. (B) Subtracted AktPH images of a growth cone before and after LY294002 (100 µM) application in the absence of the photoswitch. (C,D) Subtracted AktPH images of neurites of hippocampal neurons at stage 2. Growth-cone-like “waves” (arrowheads) traveled along the neurite anterogradely (C) or retrogradely (D). (E) Fluorescence time-lapse images of a neurite of a hippocampal neuron at stage 2 expressing Lifeact-mCh. A growth-cone-like “wave” (arrowheads) contained a markedly high concentration of F-actin. Scale bars represent 30 µm in (A) and 10 µm in (B–E).

We examined the subcellular distribution of endogenous PIP_3_ in mouse hippocampal neurons by estimating the amount of membrane-bound AktPH. AktPH exhibits two states: bound to PIP_3_ in the plasma membrane, or located in the cytoplasm (unbound to PIP_3_). We co-expressed EGFP and AktPH-mCh in hippocampal neurons, and estimated the amount of cytoplasmic AktPH from EGFP images. Then, membrane-bound AktPH images (subtracted AktPH) were produced by subtracting cytoplasmic AktPH from the original AktPH-mCh images (for details, see **Materials and Methods**). Application of the PI3K inhibitor LY294002 reduced the amount of subtracted AktPH signal in the growth cones, which verified this method of image processing (**Fig. 3B**). The subtracted AktPH signal was rich in the growth cones in both stage 2 and stage 3 neurons (**Fig. 3A**).

Growth cone-like structures, which occasionally emerged at the base of neurites, possessed high concentrations of PIP_3_ (**Fig. 3C**). These growth cone-like structures then traveled along the neurites at an average rate of 6–8 µm/min. These structures behaved similarly to “waves” [12]. We observed growth cone-like structures emerging from growth cones and traveling down neurites (**Fig. 3D**). Hereinafter, we refer to growth cone-like structures moving in either direction as “waves”. Lifeact, an F-actin marker [24] revealed that the “waves” contained a high concentration of F-actin, as previously reported [12] (**Fig. 3E**).

### Distribution and Dynamics of Endogenous PIP_3_ Inside Growth Cones

**Figure 4:**
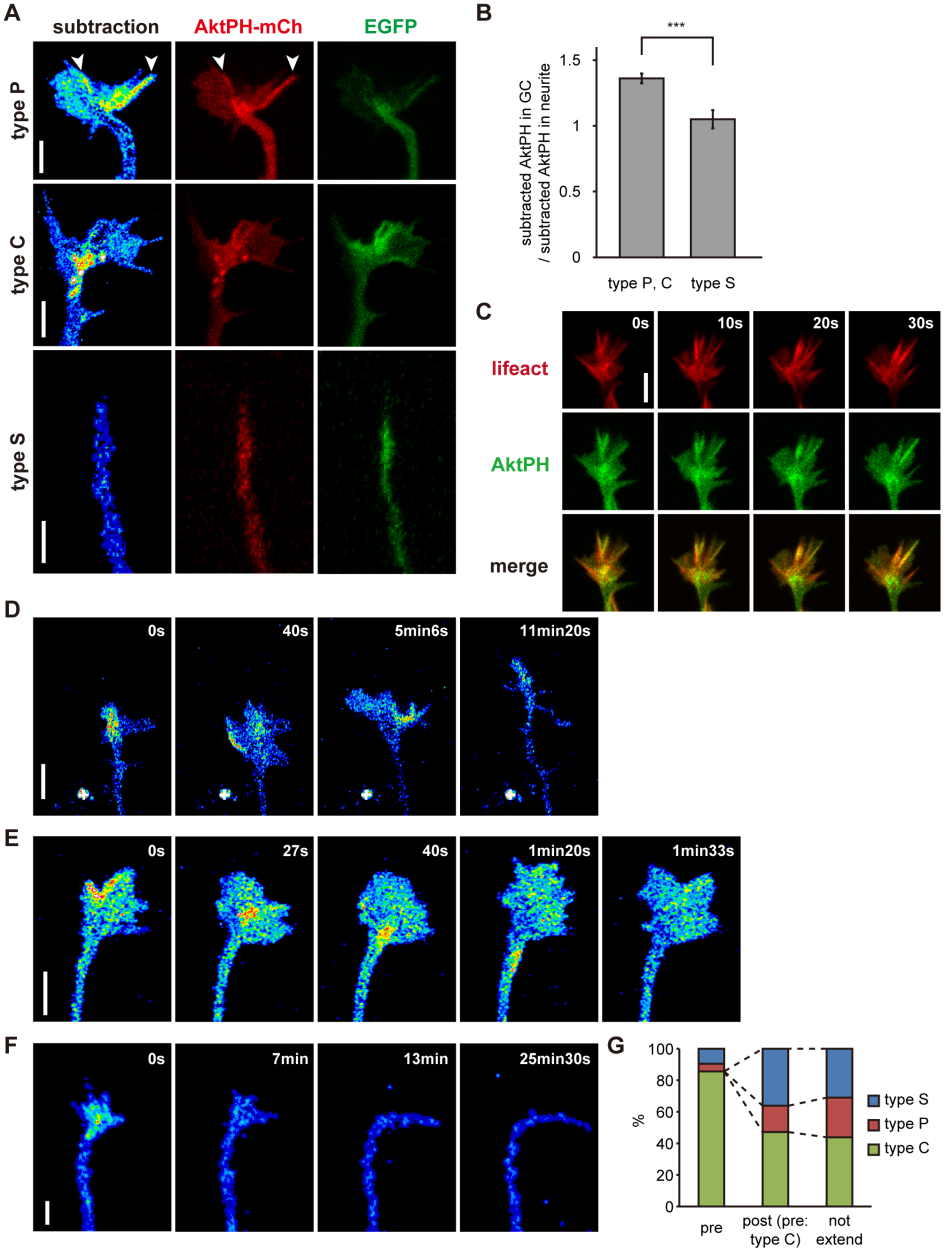
Distribution and dynamics of endogenous PIP_3_ in growth cones. (A) Subtracted AktPH (pseudo-color), AktPH-mCh (red) and EGFP (green) images of three types of growth cones in stage 2 hippocampal neurons. Arrowheads indicate PIP_3_-rich filopodia. (B) Comparison of PIP_3_ concentrations between type S (n = 64) and type P, C (n = 143) neurites. The PIP_3_ concentration in the growth cone was normalized to that in the neurite. Data are expressed as means ± s.e.m. ***P<0.0005, two sample t-test. GC stands for “growth cone”. (C) AktPH-EGFP (green), Lifeact-mCh (red) and merge time-lapse images of a growth cone of a stage 2 hippocampal neuron. (D–F) Subtracted AktPH images of growth cones of stage 2 hippocampal neurons. (D) A neurite changed its PIP_3_ distribution from type C to type P (5 min 6 s), and subsequently became type S (11 min 20 s). This neurite extended from 40 s after the start of observation to 11 min 20 s. (E) A neurite changed its PIP_3_ distribution from type P to type C (40 s), and subsequently became type P again (1 min 33 s). (F) Transition from a type P growth cone to a type S one. (G) Percentages of growth cones classified as non-extending neurites (n = 207), and neurites before (n = 42) and after extension (n = 36) showing each tip pattern (S, P and C). Only type C neurites before extension are included in “Post-extension”. Other types before extension are documented in the main text. Data were taken from time-lapse images of subtracted AktPH, and neurites were classified into “non-extending neurites” when they did not extend over 10 min. Scale bars represent 5 µm.

We noticed that the distribution of subtracted AktPH was not uniform within growth cones. Neurite growth cones are composed of a peripheral (P) domain and a central (C) domain [25]. The P domain is flat and contains actin-rich structures such as lamellipodia and filopodia [26]. The C domain contains membrane organelles that accumulate at the ends of microtubule cytoskeletons [25]. We classified the growth cones in stage 2 neurons into three types. Those with sharp tips and little flat area (<20 µm^2^ in size) were named “type S” (**Fig. 4A**). Neurite tips of this type contained a low concentration of subtracted AktPH compared with the other types (**Fig. 4B** **,** p = 0.00007, two-sample t-test). Neurite tips with large flat areas were divided into two groups based on the subtracted AktPH distribution. “Type P” neurite tips had a higher concentration of subtracted AktPH in the P domain than in the C domain, whereas “type C” neurite tips had the opposite distribution (**Fig. 4A**). Subtracted AktPH signals in the C domain were often vesicular in shape (**Fig. 4A**). Type P, C and S neurite tips comprised 30.9%, 25.1% and 44.0% of neurite tips, respectively (n = 207, mixture of stage 2 and 3 neurons) when neurites were not in the process of extension (**Fig. 4G**). In type P and C neurites, the subtracted AktPH signal was often quite strong at filopodia in the P domain of the growth cones (**Fig. 4A** **arrowheads**). The subtracted AktPH signal colocalized well with the signal for Lifeact-mCh (**Fig. 4C**).

We then conducted time-lapse observation of subtracted AktPH in neurons to examine the physiological dynamics of PIP_3_. The three types of neurite tips were not stable, but rather changed from one to another within a timescale of minutes. **Fig. 4D** shows the transition from a type C tip to a type P tip, and then to a type S tip. The level of PIP_3_ in the C domain decreased simultaneously with the increase in the surface area of the P domain (**Fig. 4D** 40 s), which suggested recycling of the PIP_3_-rich membrane. **Fig. 4E** shows the transition from a type P tip to a type C tip, and then to a type P tip again. PIP_3_ was locally accumulated in the P domain, then concentrated in the C domain (**Fig. 4E** 27 s) and retrogradely transported to the neurite (**Fig. 4E** 1 min 20 s). When the level of PIP_3_ in neurite tips decreased, those neurites simultaneously lost motility and flat area (type S) (**Fig. 4F**). On the other hand, when neurite tips acquired PIP_3_, growth cones emerged and became motile (**Fig. 2A** **and Movie S2,** neurite 1, 40 min to 99 min).

Next, we examined the relationship between PIP_3_ level and neurite extension. Neurites possessing abundant PIP_3_ in growth cones did not always extend. However, the majority of neurites (85.7%) about to extend were type C neurites. In contrast, type S neurites scarcely extended. The proportions of neurite types present when type C neurites had completed extension were 16.7%, 47.2% and 36.1% for type P, C and S neurites, respectively, which were not statistically different from those when neurites were not undergoing extension (Chi-square test, p = 0.53) (**Fig. 4G**). Type S neurites before extension (n = 4) remained type S neurites (n = 4), whereas type P neurites before extension (n = 2) became type P (n = 1) or S (n = 1) neurites when they had completed extension. Altogether, 81.0% of neurites showed reduced growth cone size and 61.9% of neurites showed reduced levels of subtracted AktPH signal during neurite extension (**Fig. 4D,F**).

### Local Activation of the PI3K Photoswitch Induces PIP_3_ Production at Neuronal Growth Cones

**Figure 5:**
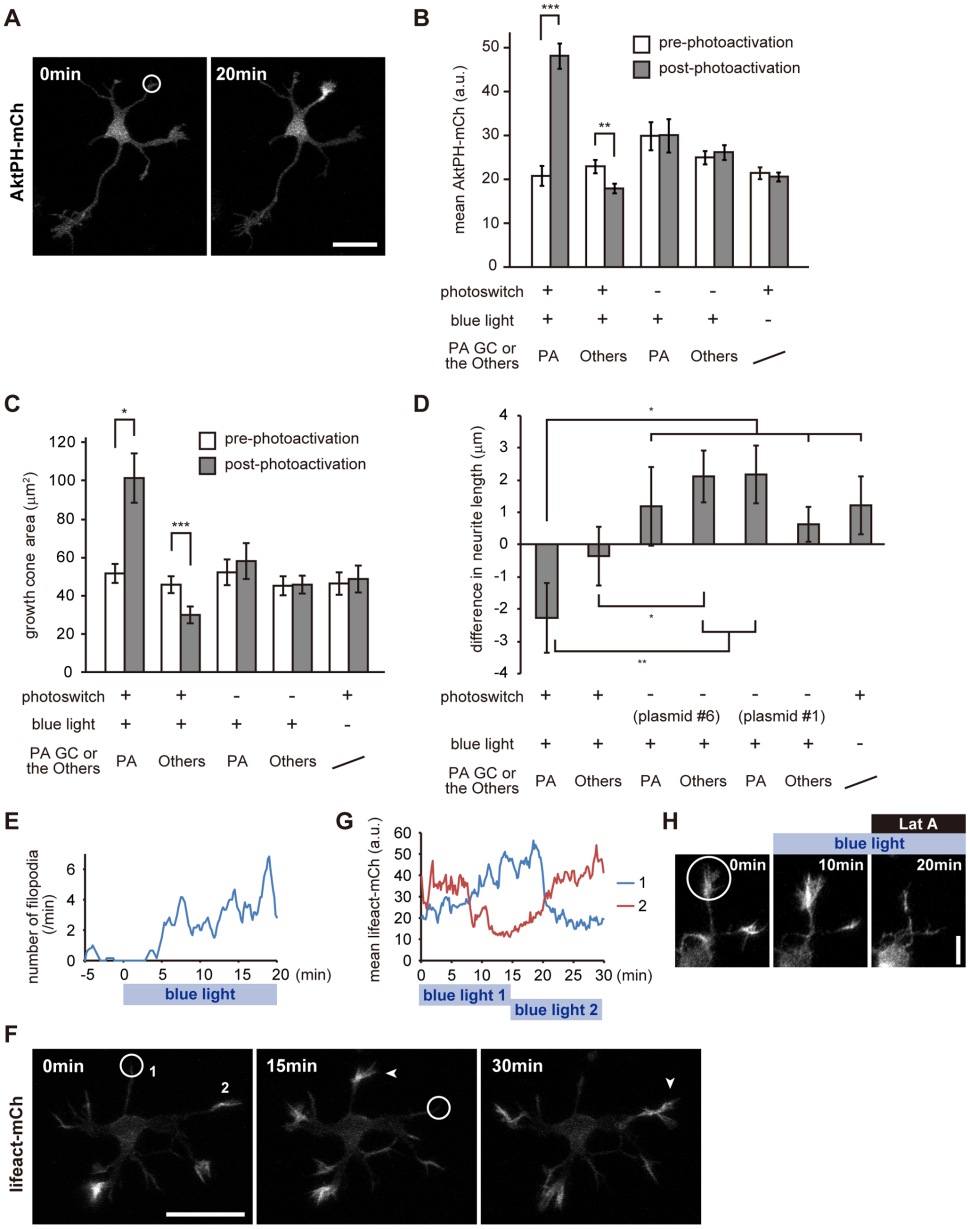
Local activation of the PI3K photoswitch in neuronal growth cones. (A) AktPH-mCh fluorescence images of a hippocampal neuron expressing plasmid #3 before (left) and 20 min after (right) photoactivation (10% of 405-nm diode laser, 0.1 Hz). (B) Mean fluorescence for AktPH-mCh in whole growth cones before and 20 min after photoactivation. (C) Sizes of growth cones before and 20 min after photoactivation. (D) Changes in neurite length 20 min after photoactivation. In this experiment, we used two negative controls, PHR-iSH deleted (plasmid #6) and iSH deleted (plasmid #1), to rule out a possible effect of photodamage. (E) Time-course of the numbers of filopodia at a photoactivated growth cone of a hippocampal neuron expressing plasmid #3. Data were taken from **Movie S5**. (F) Fluorescence images of Lifeact-mCh. A neuron expressing AktPH-mVenus, the PI3K photoswitch and Lifeact-mCh was photoactivated consecutively at two separate locations (circles), for 15 min at each location. Lifeact-mCh fluorescence increased at each photoactivated area in turn (arrowheads). (G) Time-course of change in Lifeact-mCh fluorescence at the photoactivated growth cones. Numbers represent neurites numbered in (F). (H) Fluorescence images of Lifeact-mCh. A neuron expressing AktPH-mVenus, the PI3K photoswitch and Lifeact-mCh was locally photoactivated from 0 min and subsequently Latrunculin A (Lat A, 1 µg/ml) was applied at 10 min after photoactivation. Data are expressed as means ± s.e.m. in B, C and D (10–12 neurons). *P<0.05, **P<0.005, ***P<0.0005, paired *t*-test (B, C), two sample *t*-test (D). Scale bars represent 30 µm in A and F, and 10 µm in H. GC and PA stand for “growth cone” and “photoactivated”, respectively. The white circles indicate the photoactivated area. See **Movies S4, S5 and S6**.

To study the role of PIP_3_ in growth cones, we introduced the PI3K photoswitch and AktPH-mCh (plasmid #3) into mouse hippocampal neurons. First, we photoactivated growth cones and tested whether the local activation of the photoswitch could produce PIP_3_ locally. For photoactivation, one pulse of a 405-nm diode laser (10% of the maximal intensity) was applied every 10 seconds for 20 min. This condition did not apparently damage the irradiated growth cones, because they retained motility after photoactivation (**Movie S4**). As expected, accumulation of AktPH-mCh was observed at the photoactivated growth cone in a light-dependent manner (**Fig. 5A** **, Movie S4**). We performed a similar experiment in 59 neurons expressing the PI3K photoswitch and AktPH-mCh (plasmid #3), and found that 45 of them showed localized AktPH-mCh accumulation at the irradiated growth cones. The mean fluorescence for AktPH-mCh increased 2.30±0.18-fold (n = 11) following photoactivation (**Fig. 5B**). In addition, the mean fluorescence for AktPH-mCh in the non-photoactivated growth cones in photoactivated neurons was significantly decreased (**Fig. 5B**, n = 11, p = 0.0005, paired t-test). This result indicates that local activation of the PI3K photoswitch can induce asymmetric PIP_3_ signaling between neurites.

In platelets, choresterol-enriched membrane domains were necessary for PIP_3_ production [27]. To investigate whether the choresterol-enriched membrane domains are necessary for the formation of asymmetric PIP_3_ signaling, neurons were treated with methyl-β-cyclodextrin (MβCD) to deplete choresterol and the PI3K photoswitch was locally activated. 20 min photoactivation did not change AktPH-mCh fluorescence in the presence of MβCD (**Fig. S1**). This result shows the necessity of choresterol-enriched membrane domains in the local production of PIP_3_ in neurons.

PIP_3_ could be either locally produced at or transported to growth cones. It has been reported that PIP_3_-containing vesicles were transported on microtubules by guanylate kinase-associated kinesin (KIF13B) [28]. To investigate the involvement of microtubules-based transport in the accumulation of PIP_3_ in growth cones, neurons were treated with nocodazole, a microtubules-depolymerizing agent. In neurons without the photoswitch, nocodazole treatment decreased subtracted AktPH signal at the growth cones (**Fig. S2A**). Local activation of the PI3K photoswitch at growth cones increased AktPH-mCh fluorescence in the presence of nocodazole (**Fig. S2B**). However, the degree of AktPH-mCh fluorescence increase in nocodazole-treated growth cones was about one third of that in non-treated growth cones. These results indicate that local production as well as microtubules-based transport participate in the proper accumulation of PIP_3_ in growth cones.

### Local Activation of the PI3K Photoswitch Leads to Formation of the Actin-based Active Part of Growth Cones

Next, we examined the effect of local PIP_3_ elevation on morphology of neurons. Local photoactivation of the PI3K photoswitch induced expansion of the growth cone area (**Fig. 5C**). The growth cone area after photoactivation was 2.19±0.47 times larger than that before photoactivation (n = 11), while non-photoactivated growth cones in photoactivated neurons were significantly decreased in size (p = 0.00002, paired t-test). This finding, together with the distribution of AktPH-mCh signal (**Fig. 5B**), suggests that asymmetrical PIP_3_ production induces an asymmetrical distribution of cytoplasm. PIP_3_ production in growth cones also increased the numbers of filopodia and lamellipodia, and activated their movements (**Fig. 5E** **, Movie S5**). Importantly, PIP_3_ could induce filopodia and lamellipodia in quiescent neurites (corresponding to type S), which had tapering tips without filopodia and lamellipodia, and showed no motility in time-lapse experiments (**Movie S5**).

It is widely accepted that PIP_3_ regulates actin dynamics [1], [2]. We observed the effect of local PIP_3_ elevation on actin dynamics by introducing the PI3K photoswitch and Lifeact into hippocampal neurons. Local activation of the PI3K photoswitch led to an increase in the level of F-actin at irradiated growth cones (**Fig. 5F** **, Movie S6**). The F-actin concentration began to increase shortly after photoactivation, and seemed to reach an upper threshold ∼10 min after photoactivation (**Fig. 5G**). This increase of F-actin was blocked by the application of Latrunculin A which inhibits actin polymerization (**Fig. 5H**). These results indicate that the local elevation of PIP_3_ directly induces F-actin polymerization.

PI3K photoactivation slightly shortened the irradiated neurites (**Fig. 5D**). This shortening of neurites by PI3K photoactivation was not considered to be due to photo-damage, because neurites without at least one of the photoswitch components extended in spite of the same amount of laser irradiation. We also carefully changed the photoactivation conditions by lowering laser power and changing the irradiated area as well as the temporal profile. Despite these efforts, no neurite extension was observed. The average length by which neurites were shortened (2.28 µm) was small (about one fifth of the axial length of a growth cone). This shortening could be explained by a requirement for that volume for the increase in the size of the growth cones.

### Increased PIP_3_ Signal Alone is Sufficient to Induce Growth-cone-like Structures in Ectopic Plasma Membrane Areas of Neurons

**Figure 6:**
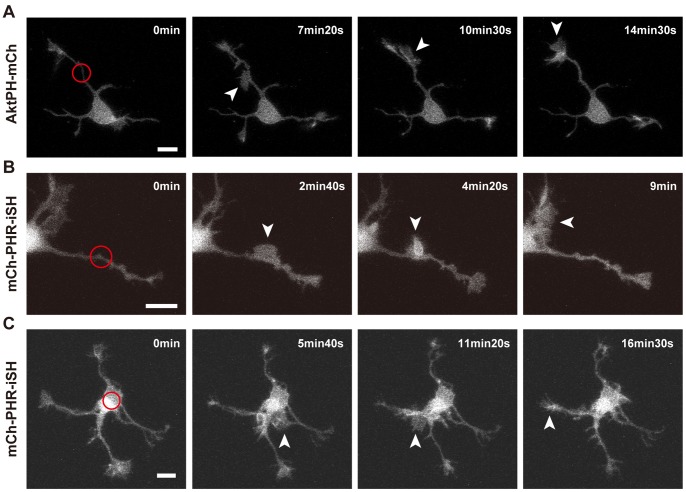
Local activation of the PI3K photoswitch in the middle of neurites or in the soma. Fluorescence images of AktPH-mCh (A) or mCh-PHR-iSH (B,C). (A,B) The middle of a neurite expressing plasmid #3 (A) or #2 (B) was photoactivated. (C) The soma of a neuron expressing plasmid #2 was photoactivated. Scale bars represent 10 µm. Arrowheads indicate “waves”. The red circles indicate the photoactivated area. See **Movie S7**.

It is of particular interest how cells respond to ectopic signals, especially in the case of signaling molecules such as PIP_3_ that are highly regulated in a spatiotemporal fashion. First, we activated the PI3K photoswitch in the middle of neurites. In five neurons, lamellipodial protrusions emerged in the photoactivated area (n = 10) (**Fig. 6A,B** **, Movie S7**). Then, the protrusions moved anterogradely (n = 3, **Fig. 6A**) or retrogradely (n = 2, **Fig. 6B** **, Movie S7**) along the neurites. The protrusions normally fused to form one growth-cone structure, and did not stay within the photoactivated area for a long time. These structures and their behavior are reminiscent of “waves” [12]. Next, the PI3K photoswitch was activated in the soma. Lamellipodial protrusions also emerged in these areas, albeit at lower frequencies (n = 2 out of 6 neurons, **Fig. 6C**). They sometimes traveled anterogradely along the neurites (**Fig. 6C**).

### PI3K Activity is Required for Rac1-induced Membrane Protrusion at Growth Cones

**Figure 7:**
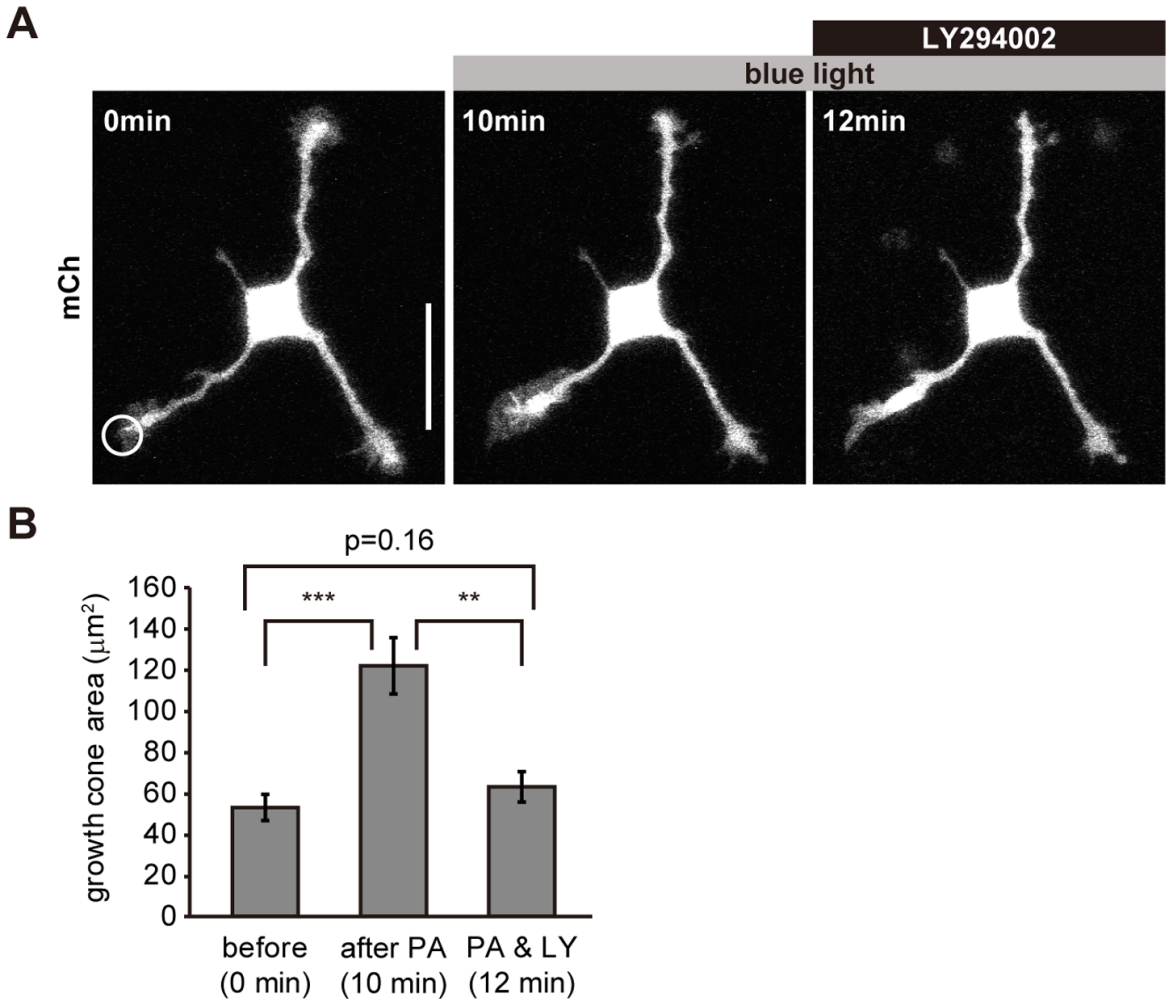
PI3K activity is required for Rac1-induced lamellipodia at growth cones. (A) Fluorescence images of a neuron expressing mCherry and PA-Rac1. The neuron was locally photoactivated (circle) and subsequently LY294002 (100 µM) was applied. (B) Size of growth cones expressing PA-Rac1 before and after photoactivation and after LY294002 (100 µM) application (n = 13). Data are expressed as means ± s.e.m. in the histograms. **P<0.005, ***P<0.0005, paired *t*-test. Scale bar represents 30 µm. See **Movie S8**.

Rac1 stimulates lamellipodia formation [29], and functions in a coordinated manner with PI3K [14]. To investigate the relationship between PIP_3_ and Rac1 in forming growth cone lamellipodia, we locally activated Rac1 using a photoactivatable form of Rac1 (PA-Rac1) [30], and tested the effect of a PI3K inhibitor on growth cones. Local photoactivation of PA-Rac1 induced a significant increase in the size of growth cones (p = 0.00004, paired t-test, n = 13) (**Fig. 7A, B** **, Movie S8**). LY294002 inhibited the motility of these growth cones and reduced their size back to their pre-photoactivation level within minutes (**Fig. 7A, B** **, Movie S8**), indicating that PI3K activity was required for the formation of growth cones.

### PIP_3_ Concentration in Growth Cones is Regulated by Endocytosis

**Figure 8:**
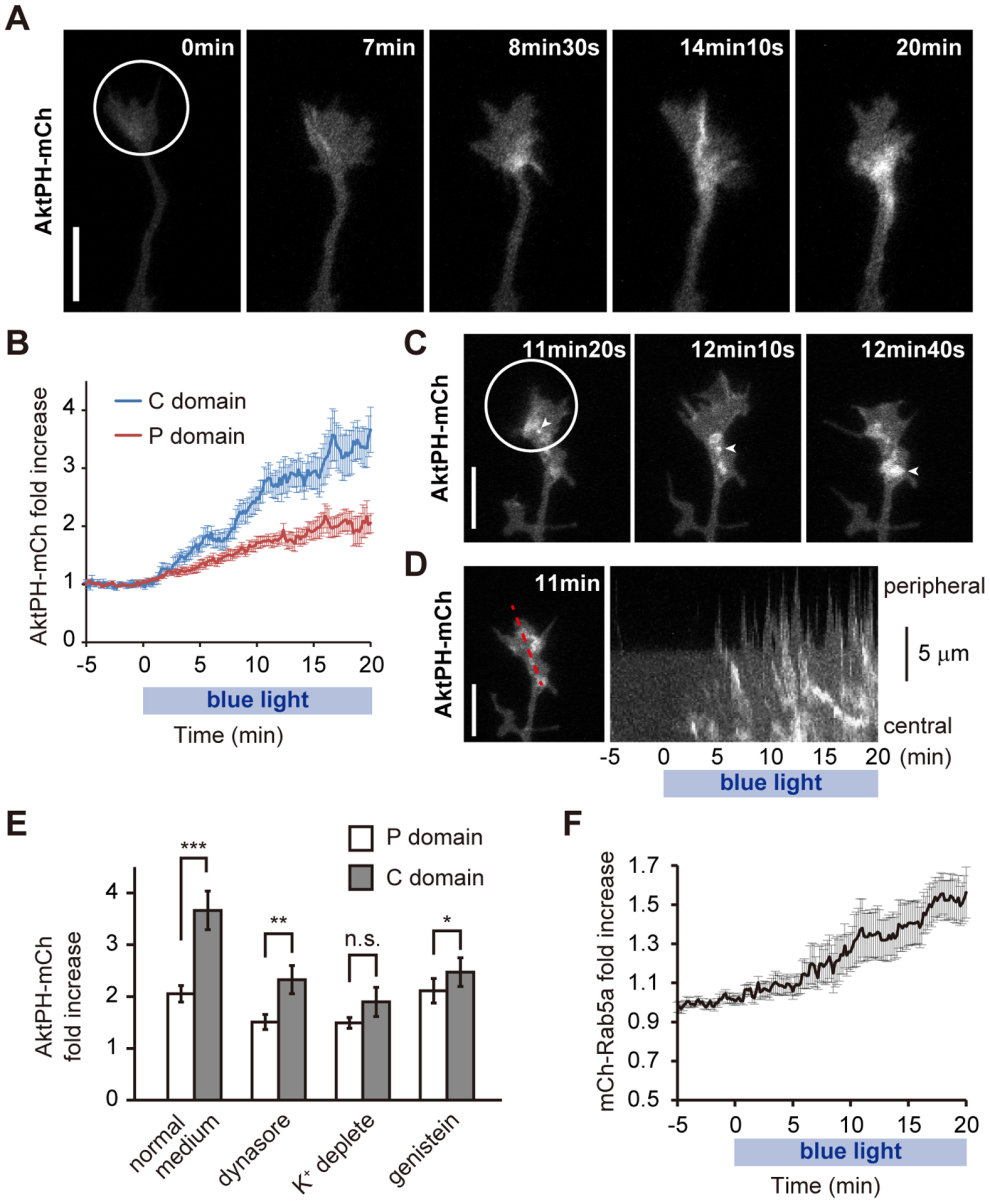
Elevated PIP_3_ is endocytosed into the C domain of growth cones. (A) High magnification of the photoactivated growth cone in **Fig. 5A**. (B) Time-course of fold changes in AktPH-mCh fluorescence in the P domain (red) and C domain (blue) of photoactivated growth cones expressing plasmid #3 (n = 11) compared with the AktPH-mCh concentration before photoactivation. We used here the same growth cones used to obtain the leftmost data in **Fig. 5B**. (C) AktPH-mCh fluorescence images of a neurite expressing plasmid #3. Arrowheads indicate the same AktPH-mCh-labeled vesicle. (D) Kymograph showing that AktPH-positive vesicles were endocytosed from the peripheral part to the central domain of the growth cone (right). The left figure indicates the red line used to take the kymograph. See **Movie S5**. (E) Effect of inhibiting endocytosis on AktPH-mCh distribution. Growth cones expressing plasmid #3 were photoactivated for 20 min with 80 µM dynasore (n = 12), in simplified buffer (see **Materials and Methods**) (n = 10), with 100 µM genistein (n = 12), or in normal imaging medium (n = 11). Fold changes in AktPH-mCh fluorescence in the P domain and C domain of photoactivated growth cones in bar graphs. n.s. stands for not statistically significant. **P<0.005, ***P<0.0005, paired *t*-test. (F) Time-course of fold changes in mCh-Rab5a fluorescence in photoactivated neurites expressing mCh-Rab5a and mVenus-PHR-iSH-2A-CIBNcaax (n = 10). Data are expressed as means ± s.e.m. The white circled areas were photoactivated. Scale bars represent 10 µm.

During photoactivation of the PI3K photoswitch, we noticed that the accumulation of AktPH-mCh was not uniform within irradiated growth cones (**Fig. 5A** **,** **8A**). After ∼8 min of photoactivation, while the AktPH-mCh level in the P domain showed a modest increase, that in the C domain of the growth cone increased drastically (**Fig. 8A**). After 20 min of photoactivation, the AktPH-mCh concentration in the C domain had increased 3.66±0.37-fold, which was significantly greater (p = 0.0003, n = 11, paired t-test) than the amount of increase in the P domain (2.05±0.16-fold) (**Fig. 8B**). Although only AktPH images were captured, this distribution of AktPH seemed to correspond to the distribution in type C neurites (**Fig. 8A**).

We often observed vesicular structures in the growth cone that were strongly labeled with AktPH-mCh during photoactivation (**Fig. 8C** **, Movie S5**). Time-lapse movies and kymographs showed that these vesicles were retrogradely transported (**Fig. 8C** **arrowheads, 8D, Movie S5**). These vesicles seemed to emerge at the sites where filopodia and lamellipodia disappeared, and their behavior was suggestive of macropinocytosis (**Movie S5**) [31]. We also tested the effect of endocytosis inhibition on PIP_3_ elevation in growth cones. Both dynasore treatment (a selective inhibitor of dynamin-dependent endocytosis) [32], K^+^ depletion (blocking clathrin-mediated endocytosis) [33], [34] and genistein treatment (inhibitor of caveolae-mediated endocytosis) [35] narrowed the difference in the size of the photoactivation-induced increase in AktPH-mCh concentrations in the P and C domains (**Fig. 8E**). Indeed, when K^+^ was depleted, the difference in AktPH-mCh concentrations between the P and C domains was not statistically significant (p = 0.17, paired t-test). This finding suggests that both clathrin-dependent and caveolae-dependent endocytosis regulates the accumulation of AktPH in the C domain of the growth cones. Interestingly, dynasore treatment and K^+^ depletion partially inhibited the photoactivation-induced increase in AktPH-mCh concentrations in both P and C domains (**Fig. 8E**). Thus, it is possible that the recycling of PIP_3_-positive endosomes is diminished by inhibition of endocytosis.

To examine whether PIP_3_ elevation increases endocytosis, the growth cones of neurons expressing the PI3K photoswitch and mCherry-tagged Rab5a were photoactivated. Because Rab5 mediates fusion of endocytotic vesicles to form early endosomes [36], mCh-Rab5a fluorescence on early endosomes should be much brighter than that in plasma membranes. Activation of the PI3K photoswitch increased the fluorescence of mCh-Rab5 in photoactivated growth cones and neurites (**Fig. 8F** **, Movie S9**), suggesting that PIP_3_ elevation facilitates endocytosis at growth cones.

**Figure 9:**
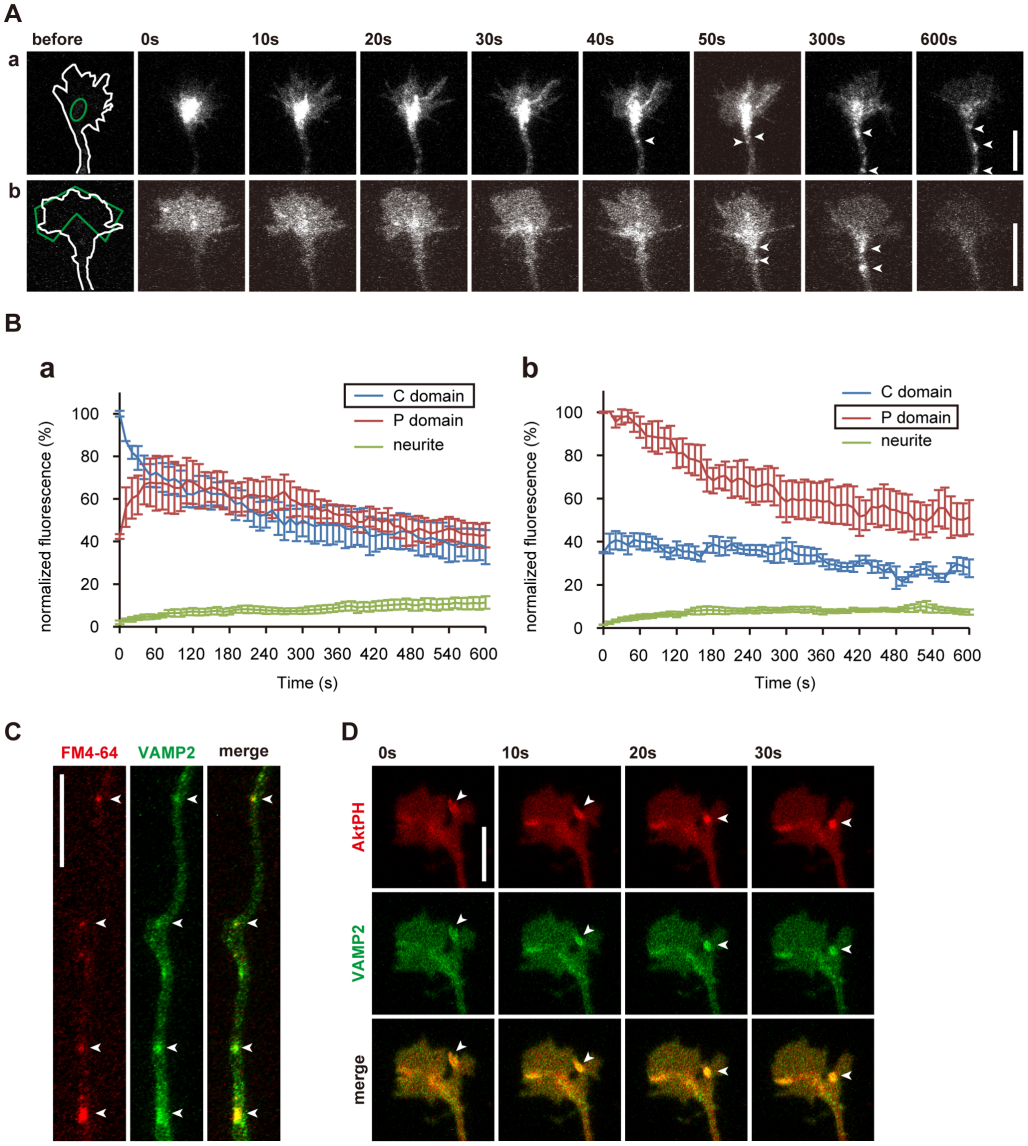
PIP_3_ is endocytosed via a constitutive endocytotic pathway at growth cones. (A) PA-GFP-VAMP2 was photoactivated (green line) at either the C domain (a) or the P domain (b) of the growth cone. Arrowheads indicate retrogradely transported vesicles. (B) Time-course of changes in VAMP2 fluorescence in the P and C domains of growth cones and small areas of neurites. The C or P domain was photoactivated in (a) or (b), respectively (n = 6 in a, n = 8 in b). VAMP2 moved both from the C to the P domain and from the P to the C domain. VAMP2 level in the growth cone gradually decreased, which reflects the retrograde transport observed in (A). Graphs are expressed as means ± s.e.m. (C) Axon of a stage 3 hippocampal neuron expressing PA-GFP-VAMP2 (green) and loaded with FM4-64 (red). PA-GFP-VAMP2 colocalized with FM4-64 (arrowheads). (D) Time-lapse images of distal part of a neurite of a stage 2 neuron expressing both PA-GFP-VAMP2 (green) and AktPH-mCh (red). An AktPH-mCh-labeled vesicle colocalized with fluorescent VAMP2 (arrowheads). Scale bars represent 10 µm.

To further confirm the endocytotic retrieval of PIP_3_-rich membranes, we examined the dynamics of a membranous protein known to be endocytosed at nerve terminals [37]. Neurons were co-transfected with vectors for vesicle-associated membrane protein 2 (VAMP2) tagged with photoactivatable (PA-) GFP [38] and mCh. mCh was used to visualize the shapes of neurons before photoactivating PA-GFP. Either the P domain or the C domain of the growth cone was photoactivated using a 405-nm laser, and time-lapse images were acquired immediately afterwards. When the C domain was photoactivated (**Fig. 9A** **a, Ba**), vesicular structures in the C domain were retrogradely transported from 40 s after photoactivation (**Fig. 9A** **a**). In contrast, when the P domain was photoactivated, fluorescent VAMP2 in the P domain was gradually incorporated into the C domain (**Fig. 9A** **b,Bb**). Later (50–600 s after photoactivation), large, highly fluorescent vesicles in the C domain increased in number and moved into neurites (**Fig. 9A** **b, arrowheads**).To study the nature of these vesicles, we labeled endosomes by loading a red marker for endocytosis (FM4-64). FM4-64 colocalized with fluorescent VAMP2 (**Fig. 9C**). In addition, AktPH-labeled vesicles often colocalized and moved together with VAMP2-labeled vesicles (**Fig. 9D**). These observations confirmed that PIP_3_-rich membranes were endocytosed.

## Discussion

PIP_3_ regulates various cellular functions via its intracellular distribution [1]. However, it is difficult to understand the local primary functions of PIP_3_ because of the lack of techniques for manipulating PIP_3_ in a spatiotemporal manner. In this study, we addressed this issue by using a PI3K photoswitch that could induce spatiotemporal production of PIP_3_ upon blue light exposure (**Fig. 5**). We found that PIP_3_ regulated the size and motility of growth cones (**Fig. 5**), and that PIP_3_ induced growth-cone-like “waves” (**Fig. 6**). Additionally, we found that endocytosis is used as a novel mechanism for regulating PIP_3_ signaling in growth cones (**Figs. 8** **,** **9**). Our study exemplifies the power of optogenetics for dissecting the functions of signaling molecules such as PIP_3_ under tight spatiotemporal regulation.

### Model for the Relationship between PIP_3_ Dynamics and Growth Cones

**Figure 10:**
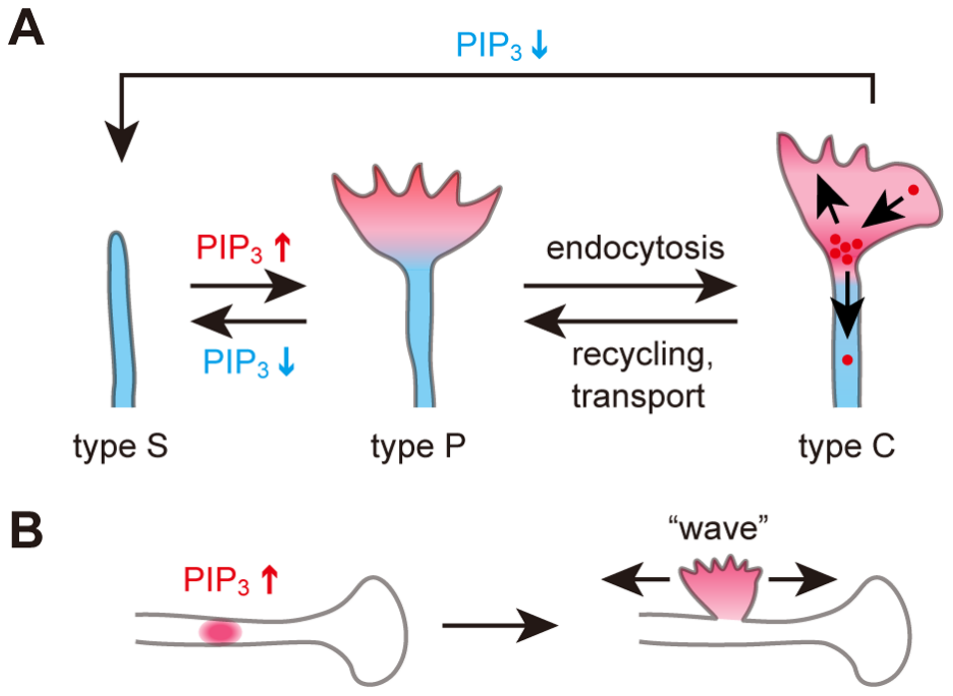
Model for PIP_3_ dynamics and functions in developing neurons. (A) Type S neurite possesses little PIP_3_ (left). When the PIP_3_ concentration increases, the neurite gains a growth-cone protrusion and becomes type P (center). PIP_3_ is endocytosed from the P domain to the C domain. This converts the neurite to type C (right). When endocytosed PIP_3_ vesicles are recycled into the P domain or retrogradely transported, the neurite returns to being type P. A decrease in PIP_3_ concentration transforms the neurite from type P, C to type S. (B) Mechanism underlying the formation of growth-cone-like “waves”.

Our findings led us to propose the following model for growth cone dynamics (**Fig. 10A**). When PI3K is activated intracellularly, or by other mechanisms such as extracellular growth factors, the PIP_3_ level increases to form an active part of a growth cone. PIP_3_-containing vesicles may also play a role in this process. First, PIP_3_ resides in the P domain of the growth cone (type P). When PIP_3_ is further elevated, PIP_3_ is endocytosed into the C domain (type C) (**Figs. 8** **,** **9**). The endocytosed PIP_3_ vesicles are either transported retrogradely (**Fig. 8**), or are recycled to form the P domain (**Figs. 4** **,** **8**) [4] or stay in the C domain. When the number of these vesicles or PIP_3_ on these vesicles has decreased, the growth cone becomes type P again. Once the amount of PIP_3_ in the growth cone has decreased further to a level similar to that in the plasma membrane of the neurite, the neurite loses its growth cone and becomes type S again.

### Intracellular Function of PIP_3_ in Neurons

Previously, it was shown that PI3K activation is essential for membrane expansion at the growth cone [39] and that PI3K accumulates in the tips of neurites [40]. We directly showed that PIP_3_ in growth cones induces actin-rich active structures such as filopodia and lamellipodia, and increases the size and motility of growth cones (**Fig. 5**). Also, PI3K activity was necessary (**Fig. 7**) and sufficient (**Movie S5**) for membrane expansion at the growth cone. These results suggest that PIP_3_ is the master regulator of the active parts of growth cones.

One of the advantages of optogenetics is that it enables particular molecules at specific locations to be activated. We elevated PIP_3_ in the middle of neurites and on the neuronal soma to find that PIP_3_ alone induces growth-cone-like “waves” (**Fig. 6**). Also, the “waves” contained abundant PIP_3_ (**Fig. 3**). Although “waves” were first documented over 10 years ago and were considered to be important for neurite elongation and branching [41], [42], the molecular mechanisms underlying the formation of “waves” has remained elusive. In our model, local PIP_3_ elevation underlies the formation of “waves” (**Fig. 10B**). This schema provides a unified view of the origins of “waves” and growth cones. It is consistent with reports that “waves” have similar characteristics to growth cones such as movement, ultrastructure and the molecules they contain [12], [41]. PIP_3_ did not seem to determine whether the “waves” moved anterogradely or retrogradely (**Fig. 3** **,** **6**), specification of which may require other mechanisms.

The role of PIP_3_ in neurite extension is obscure. PIP_3_ elevation did not induce neurite elongation (**Fig. 5**), but the majority of neurites before extension were type C (**Fig. 4**). Considering that local accumulation of PIP_3_ induced asymmetric accumulation of cytoplasm (**Fig. 5**), PIP_3_ might prepare neurites for extension by stimulating accumulation of proteins and membranes in growth cones, which might then be used for building the extended parts of the neurites.

It was recently reported that local activation of G protein-coupled receptor (GPCR) signaling induced PIP_3_ elevation, which led to neurite initiation and extension when the photoactivated area was moved [43]. The discrepancy between those findings and our results (**Fig. 5**) could have resulted from downstream effectors of GPCR signaling other than PIP_3_, such as JNK or ERK1/2 [44].

### Novel Regulatory Mechanisms of PIP_3_ in Growth Cones

We found that PIP_3_-rich membranes were endocytosed (**Figs. 8** **,** **9**). PIP_3_ elevation induced by the PI3K photoswitch did not lead to a large increase in PIP_3_ concentration in the P domain of growth cones (**Fig. 8**). Considering that the P domain is flat and PIP_3_ acts on plasma membranes, endocytosis may restrict the PIP_3_ concentration in the plasma membranes of growth cones. This notion is also supported by the finding that PIP_3_ elevation facilitated endocytosis (**Fig. 8**). Although PIP_3_ may still perform some roles within endocytosed vesicles [4], [45], PIP_3_ is primarily localized within the plasma membrane where it recruits downstream signaling effectors [2]. PIP_3_ on endosomal structures is considered unable to contribute to the rapid changes in the size and motility of growth cones, which are strictly dependent on the actin cytoskeleton underneath the plasma membrane.

## Materials and Methods

### DNA Constructs

CRY2PHR-mCherry and CIBN-pmGFP were kind gifts from Chandra Tucker. EYFP-C1-p85β was purchased from Addgene (Plasmid #1408). PH-mRFP1 (the PH domain of human Akt1) was a generous gift from Daniel F. Cimino. We introduced a silent mutation into PH-mRFP by overlap PCR to delete the NotI site in the original sequence.

To make CAG promoter-controlled backbone plasmids, a CAG promoter was cut out from the adenovirus cosmid vector (TaKaRa) and subcloned into the CMV promoter region of pEGFP-N1 and pEGFP-C1 vectors (Clontech). We termed them CAG-EGFP-N1 and CAG-EGFP-C1, respectively. Other color variations of these plasmids were made by subcloning PA-GFP, mCherry and mVenus into the EGFP site.

Some plasmids were designed to contain the Woodchuck Hepatitis Virus Posttranscriptional Regulatory Element (WPRE) in the 3′ untranslated region of coding sequences to enhance protein expression [46]. Note that the insertion of WPRE does not change the amino acid sequence.

AktPH (human Akt1, aa.1–164) and mCherry were linked by DPPVAT (amino acid sequence; the same shall apply hereinafter). CIBN (*Arabidopsis thaliana* cryptochrome-interacting basic-helix-loop-helix 1, aa.1–170) and the CAAX membrane targeting sequence from K-ras (KKKKKKSKTKCVIM) were linked by LYKG. In plasmids #1 and #2, mCherry and CRY2PHR (*A. thaliana* CRY2, aa.1–498) were linked by SGLRSRAQASNSAVDGT. The linker SAGGSAGGSAGGPRA was inserted between CRY2PHR and p85β iSH (mouse p85β aa.420–615). The 2A peptide sequence (EGRGSLLTCGDVEENPGP) was followed by the linker APGT or APGS to locate the 2A product protein to the precise subcellular domain [47]. PHR-2A, iSH-2A and mCherry-2A contained no peptide linker. mVenus-PHR-iSH-2A-CIBNcaax and AktPH-mVenus-2A-PHR-iSH-2A-CIBNcaax are the same as plasmid #2 and #3, respectively, except that mCherry is replaced with mVenus. All of the plasmids shown in **Fig. 1B** were under the control of the CAG promoter.

pTriEx-mVenus-PA-Rac1 [30] was purchased from Addgene (plasmid #22007) and was amplified by PCR to make mCherry-2A-PA-Rac1. The peptide linker between 2A and PA-Rac1 was APGS. These plasmids were also under the control of the CAG promoter.

Lifeact [24] was amplified by overlap PCR of two oligos (5′-CCCAAGCTTGCCACCATGGGCGTGGCCGACCTGATCAAGAAGTTCGAGA-3′ and 5′-TTTGGATCCCCTTCCTCCTTGCTGATGCTCTCGAACTTCTTGATCAGGT-3′) and was inserted into the HindIII–BamHI sites of mCherry-N1.

VAMP2 cDNA was amplified by PCR and cloned into the XhoI and EcoRI sites of PA-GFP-C1 (kind gift from Jennifer Lippincott-Schwartz). Then, PA-GFP-VAMP2 was subcloned into the NheI and EcoRI sites of CAG-EGFP-C1-WPRE.

To make CAG-K381-EGFP-WPRE, K381-EYFP [22] was subcloned into the EcoRI–BamHI sites of EGFP-N1 (Clontech); then, K381-EGFP was subcloned into the EcoRI–NotI sites of CAG-EGFP-N1-WPRE.

Rab5a cDNA was amplified from EGFP-Rab5a by PCR and cloned into the SalI and NotI sites of CAG-mCherry-C1.

All coding regions were verified by sequencing.

### HEK293 Cell Culture and Transfection

HEK293 cells were maintained in MEM (minimal essential medium; Nacalai Tesque) supplemented with 10% (v/v) fetal bovine serum, penicillin (50 U/ml), and streptomycin (50 mg/ml) (MEM-FBS). Cells were passaged every 2–3 days. Two days before experiments, cells were seeded on matrigel- or poly-L-lysine-coated glass-bottomed dishes (Greiner) and grown close to confluency. The next day, plasmid transfection was performed using Lipofectamine 2000 (Invitrogen) according to the manufacturer’s instructions. Cells were maintained in MEM-FBS overnight after transfection. On the experimental day, cells were starved in serum-free MEM for ≥3 hours so as not to activate endogenous PI3K by exogenous factors. Then, the medium was replaced with pre-warmed L-15 medium (Invitrogen) before imaging. Human EGF and LY294002 were purchased from PeproTech and Cell Signaling Technology, respectively.

### Primary Culture and Electroporation of Hippocampal Neurons

Primary hippocampal neurons derived from mouse embryos were cultured following the method of Kaech and Banker [48] with some modifications. In brief, glass coverslips (Hecht Assistent, #1001-18, No.1) for hippocampal neurons were bathed for ≥24 hours in nitric acid (≥65%), rinsed four times in distilled water and sterilized at 225°C for 10 hours. Coverslips were then sealed onto 14-mm-holed 35-mm Petri dishes with silicone grease and coated with poly-L-lysine (1 mg/ml in borate buffer, pH 8.5) overnight at room temperature. Coverslips were washed twice with distilled water for 2 hours to remove excess poly-L-lysine (Nacalai Tesque, 28360-14, MW 30,000–70,000) and subsequently incubated with N-MEM (MEM supplemented with 1 mM pyruvic acid and 0.6% glucose) containing 10% horse serum in 5% CO_2_ at 37°C overnight. E16.5 mice (purchased from Sankyo Lab Service, Japan) were killed by cervical dislocation. The procedure was done quickly and expertly to ameliorate suffering. Then, the hippocampi of the mice were dissected, trypsinized for 15 min with 0.5% trypsin-EDTA at 37°C and washed three times in HBSS for 5 minutes. After removing HBSS, cells were resuspended in R buffer (Neon electroporation kit, Invitrogen), pipetted 10–15 times with a 1000-µl pipette tip and 4–5 times with a 200-µl pipette tip or a fire-polished glass Pasteur pipette, mixed with the DNA constructs, electroporated using the Neon electroporation system (Invitrogen) and plated onto the dishes described above. For electroporation, pulse voltage, pulse width and pulse number were set to 1700 V, 20 ms and once, respectively. The cells were kept in 5% CO_2_ at 37°C. After 2 hours, the medium was exchanged to N-MEM containing B27 (Invitrogen) and the cells were brought back to the CO_2_ incubator without astrocyte co-culture. In this condition, stage 2–3 transition typically occurs 24–48 hours after plating. To prevent cells from being unintentionally exposed to blue light, dishes were covered with aluminum foil when incubated. The medium was replaced with L-15 supplemented with B27 before imaging.

### Ethics Statement

All procedures relating to the care and treatment of the animals were performed in accordance with the National Institutes of Health (NIH) guidelines. All animal experiments were approved by the Institutional Animal Care and Use Committee of Tokyo Medical and Dental University (0130270C).

### Live-cell Imaging and Photoactivation

Time-lapse images were obtained using confocal laser scanning microscopy (Bio-Rad MRC-1024, Bio-Rad Laboratories) on an Axiovert100 inverted microscope equipped with a Kr/Ar laser (λ = 488 nm, 568 nm, Zeiss), a HeCd laser (λ = 442 nm, KIMMON Electric Co., Ltd.) and a 40× C-Apochromat objective lens (Zeiss, numerical aperture = 1.2) or using LSM510 META laser scanning microscopy (Zeiss) on an Axiovert 200M microscope (Zeiss) equipped with a diode laser (λ = 405 nm, 25.0 mW), a multi-line argon gas laser (λ = 458 nm, 477 nm, 488 nm, 514 nm, 30.0 mW), a HeNe laser (λ = 543 nm, 1.0 mW) and a 40× C-Apochromat objective lens (Zeiss, numerical aperture = 1.2). Whole cell activation experiments in HEK293 cells were typically performed by scanning the whole area with the HeCd laser (3%) simultaneously with image acquisition (5-s intervals) using the MRC1024 system. For local photoactivation in hippocampal neurons, diode laser light (λ = 405 nm) was focused on the selected area of the neuron (15–22.5 µm in diameter), which was irradiated typically every 10 s using the LSM510 META system. A comparison of different laser activations is shown in **Fig. 1I**. Some images of neurons expressing both AktPH-mCh and EGFP were taken using a 63× C-Apochromat objective lens (Zeiss, numerical aperture = 1.2). Latrunculin A, nocodazole and MβCD were purchased from Cayman chemical, Sigma and Wako, respectively.

### FM4-64 Loading

For FM4-64 labeling, electroporated hippocampal neurons at 1–2 DIV were treated with 15 µM FM4-64 (Molecular Probes, Inc., Eugene, OR) cultured in high potassium Ringer solution (31.5 mM NaCl, 90 mM KCl, 2 mM CaCl_2_, 2 mM MgCl_2_, 30 mM glucose, 25 mM HEPES, pH 7.3) [49], [50] for 10 min, and then washed three times over a 15-min period with L-15. The medium was replaced with L-15 supplemented with B27 before imaging.

### Inhibition of Endocytosis

For the dynasore and genistein experiments, hippocampal neurons at 1–2 DIV were imaged with L-15 supplemented with B27 and either 80 µM dynasore (Merck) or 100 µM genistein (Wako). To deplete K^+^, neurons were washed once with L-15, and treated with hypotonic medium (N-MEM/H_2_O, 1∶1) for 5 min. Neurons were then incubated with simplified buffer (140 mM NaC1, 10 mM KCl, 1 mM CaCl_2_, 1 mM MgCl_2_, 20 mM HEPES, 5.5 mM glucose, and 1% (vol/vol) BSA, pH 7.4) [33] for 30 min, and were imaged without medium change.

### Image Analysis

Image analysis was performed using ImageJ software (NIH).

#### Membrane translocation assay

The membrane translocations of mCh-PHR, mCh-PHR-iSH and AktPH-mCh shown in **Fig. 1** were evaluated based on changes in cytoplasmic fluorescence [13]. Fluorescent signal intensity was measured in a 2.25 µm×2.25 µm square region in the cytoplasm throughout imaging. Then, the relative translocation level at time-point X was calculated by dividing the fluorescence intensity at time-point X by the average fluorescence intensity before photoactivation.

#### Measurement of fluorescence and the sizes of growth cones in neurons

The AktPH-mCh level and the size of the P domain of growth cones were calculated by subtracting those of the C domain from those for the whole growth cone. The AktPH-mCh concentration was calculated by dividing AktPH-mCh level by the size of the corresponding area.

#### Membrane dynamics measured by PA-GFP-VAMP2 in growth cones

Neurons were co-transfected with PA-GFP-VAMP2 and mCherry to visualize neurons prior to photoactivation. The P or C domain of growth cones was chosen and irradiated by 5–10 pulses of maximal intensity with a 405-nm diode laser, followed by image acquisition. The P domain and the C domain of the growth cone were determined manually. The level of GFP fluorescence was normalized to the initial fluorescence intensity in the irradiated area immediately after photoactivation.

#### Subtraction image of AktPH

We generated subtraction images of AktPH in **Figs. 3** **,** **4** by the following procedures. Neurons were transfected with AktPH-mCh and EGFP. We estimated the cytosolic fraction of AtkPH-mCh assuming that cytoplasmic proteins would distribute similarly to EGFP. EGFP images were multiplied by the constant k, so as to represent the distribution of cytosolic (unbound to PIP_3_) AktPH-mCh. Values of k for each cell were determined as the ratio of mean fluorescence for AktPH-mCh to that of EGFP in a part of the neurite shaft where membrane-bound AktPH-mCh (bound to PIP_3_) was considered to be scarce. k-multiplied EGFP images were subtracted from AktPH-mCh images. Then, upper thresholds were set as pseudo-color images with a high dynamic range. Images were subjected to a smoothing filter, followed by binary masking [51].

#### Statistics

Data are expressed as means ± s.e.m. Statistical analyses were performed using Excel software (Microsoft) or manually. *P* values <0.05 were judged statistically significant.

## Supporting Information

Figure S1 **Choresterol-enriched membrane domains are necessary for local accumulation of PIP_3_.** Time-course of fold changes in AktPH-mCh fluorescence in photoactivated growth cones expressing plasmid #3. Growth cones expressing plasmid #3 were photoactivated for 20 min with 5 mM MβCD (n = 11) or in normal imaging medium (n = 11, the same growth cone as the leftmost data in Fig. 5B). Data are expressed as means ± s.e.m.
(TIF)

Figure S2 **Microtubules-based transport is partially involved in the production of PIP_3_ at growth cones.** (A) Subtracted AktPH images of a growth cone before and after nocodazle (3.3 µM) application in the absence of the photoswitch. (B) Time-course of fold changes in AktPH-mCh fluorescence in photoactivated growth cones expressing plasmid #3. Growth cones expressing plasmid #3 were photoactivated for 20 min with 3.3 µM nocodazole (n = 18) or in normal imaging medium (n = 11, the same growth cone as the leftmost data in Fig. 5B). Data are expressed as means ± s.e.m.
(TIF)

Movie S1 **AktPH-mCh repeatedly moved to the plasma membrane upon photoactivation of the PI3K photoswitch.** Time-lapse images of a HEK293 cell expressing AktPH-mCh and the PI3K photoswitch (plasmid #3). AktPH-mCh fluorescence images were acquired every 10 s. Photoactivation was given to whole cell with one pulse of a 442-nm HeCd laser (3%) at 0 min, 15 min and 30 min. Repeated activation successfully translocated AktPH-mCh to the plasma membrane.
(MOV)

Movie S2 **Dynamics of PIP_3_ signaling during the formation of axon.** Time-lapse images of a mouse hippocampal neuron expressing AktPH-mCh (pseudo-color, left) and K381-EGFP (green, light). Fluorescence images were taken every 1 min. The AktPH-mCh concentration in neurites varied on a timescale of minutes. Note that the K381 level did not correlate with the AktPH-mCh level. Selected images from this movie are shown in **Fig. 2A**.
(MOV)

Movie S3 **Dynamics of PIP_3_ signaling during the formation of axon.** Time-lapse images of a mouse hippocampal neuron expressing AktPH-mCh (pseudo-color, left) and K381-EGFP (green, light). Fluorescence images were taken every 5 min. The AktPH-mCh level in the future axon (neurite 4), which did not have a lamellipodial growth cone, was often lower than that in neurite 3. Selected images from this movie are shown in **Fig. 2B**.
(MOV)

Movie S4 **Local production of PIP_3_ by activation of the PI3K photoswitch.** Time-lapse images of a stage 2 mouse hippocampal neuron expressing AktPH-mCh and the PI3K photoswitch (plasmid #3). AktPH-mCh fluorescence images were acquired every 10 s and showed in pseudo-color. The red circled areas were continuously photoactivated (405-nm diode laser at 10% of maximal power, 0.1 Hz for 20 min per growth cone) from 0 min. The photoactivated growth cone was changed at 20 min. Photoactivation of the PI3K photoswitch significantly increased AktPH-mCh fluorescence and the area of the growth cone in a light-dependent manner.
(MOV)

Movie S5 **PIP_3_ production induced filopodia and lamellipodia, and endosomes labeled with AktPH-mCh.** Time-lapse images of a growth cone of a stage 2 mouse hippocampal neuron expressing AktPH-mCh and the PI3K photoswitch (plasmid #3). AktPH-mCh fluorescence images were acquired every 10 s. The red circled area was continuously photoactivated (405-nm laser at 10% of maximal power, 0.1 Hz for 20 min) from 0 min. Photoactivation of the PI3K photoswitch converted quiescent neurite to motile one with highly active filopodia and lamellipodia. It also significantly increased the area of the growth cone and the emergence of AktPH-labeled vesicles. Selected images from this movie are shown in Fig. 8C,D.
(MOV)

Movie S6 **Local PIP_3_ production increased F-actin at growth cones.** Time-lapse images of a stage 2 mouse hippocampal neuron expressing Lifeact-mCh, AktPH-mVenus, and the PI3K photoswitch. Lifeact-mCh fluorescence images were acquired every 10 s. The red circled areas were continuously photoactivated (405-nm laser at 10% of maximal power, 0.1 Hz) from 0 min. The photoactivated growth cone was changed at 15 min. Photoactivation of the PI3K photoswitch increased Lifeact-mCh fluorescence and growth cone motility. Selected images from this movie are shown in **Fig. 5F**.
(MOV)

Movie S7 **Local PIP_3_ production in the middle of the neurites caused “wave”.** Time-lapse images of a stage 2 mouse hippocampal neuron expressing the PI3K photoswitch (plasmid #2). mCh-PHR-iSH images were acquired every 10 s. The red circled area was continuously photoactivated (405-nm laser at 10% of maximal power, 0.1 Hz) from 0 min. Photoactivation of the PI3K photoswitch induced accumulation of mCh-PHR-iSH. Then, lamellipodial protrusions formed at the photoactivation site and moved retrogradely. Selected images from this movie are shown in **Fig. 6B**.
(MOV)

Movie S8 **Inhibition of PI3K activity blocked the PA-Rac1-induced formation of growth cone lamellipodia.** Time-lapse images of a stage 2 mouse hippocampal neuron expressing both mCherry and PA-Rac1. mCherry fluorescence images were acquired every 10 s. The red circled area was continuously photoactivated (405-nm laser at 10% of maximal power, 0.1 Hz) from 0 min and LY294002 (100 µM) was applied at 10 min. LY294002 application blocked formation of growth cone lamellipodia induced by PA-Rac1 photoactivation and prevented growth cone motility. Selected images from this movie are shown in **Fig. 7A**.
(MOV)

Movie S9 **PIP_3_ elevation increased the number of Rab5a-positive endosomes.** Time-lapse images of a growth cone of a stage 2 mouse hippocampal neuron expressing mCh-Rab5a and mVenus-PHR-iSH-2A-CIBNcaax. mCh-Rab5a fluorescence images were acquired every 10 s. The red circled area was continuously photoactivated (405-nm laser at 10% of maximal power, 0.1 Hz) from 0 min. Accumulation of Rab5a-positive vesicles increased in the C domain of the growth cone and in neurites in response to photoactivation.
(MOV)

## References

[1] Vanhaesebroeck B, Guillermet-Guibert J, Graupera M, Bilanges B (2010) The emerging mechanisms of isoform-specific PI3K signalling. Nat Rev Mol Cell Biol 11: 329–341. 20379207 10.1038/nrm2882

[2] Saarikangas J (2010) Regulation of the actin cytoskeleton-plasma membrane interplay by phosphoinositides. Physiol Rev 90: 259–289. 20086078 10.1152/physrev.00036.2009

[3] Janetopoulos C, Firtel R a (2008) Directional sensing during chemotaxis. FEBS Lett 582: 2075–2085. 18452713 10.1016/j.febslet.2008.04.035 PMC2519798

[4] Fields IC, King SM, Shteyn E, Kang RS, Fo H, et al.. (2010) Phosphatidylinositol 3,4,5-trisphosphate localization in recycling endosomes is necessary for AP-1B-dependent sorting in polarized epithelial cells. Mol Biol Cell. 10.1091/mbc.E09-01-0036 PMC2801725 19864464

[5] Ming G, Song H, Berninger B, Inagaki N, Tessier-lavigne M, et al (1999) Phospholipase C-gamma and phosphoinositide 3-kinase mediate cytoplasmic signaling in nerve growth cone guidance. Neuron 23: 139–148. 10402200 10.1016/s0896-6273(00)80760-6

[6] Henle SJ, Wang G, Liang E, Wu M, Poo M-M, et al (2011) Asymmetric PI(3,4,5)P3 and Akt signaling mediates chemotaxis of axonal growth cones. J Neurosci 31: 7016–7027. 21562263 10.1523/JNEUROSCI.0216-11.2011 PMC3133771

[7] Ketschek A, Gallo G (2010) Nerve growth factor induces axonal filopodia through localized microdomains of phosphoinositide 3-kinase activity that drive the formation of cytoskeletal precursors to filopodia. J Neurosci 30: 12185–12197. 20826681 10.1523/JNEUROSCI.1740-10.2010 PMC2944214

[8] Shi S-H, Jan LY, Jan Y-N (2003) Hippocampal neuronal polarity specified by spatially localized mPar3/mPar6 and PI 3-kinase activity. Cell 112: 63–75. 12526794 10.1016/s0092-8674(02)01249-7

[9] Ménager C, Arimura N, Fukata Y, Kaibuchi K (2004) PIP3 is involved in neuronal polarization and axon formation. J Neurochem 89: 109–118. 15030394 10.1046/j.1471-4159.2004.02302.x

[10] Yoshimura T, Arimura N, Kawano Y, Kawabata S, Wang S, et al (2006) Ras regulates neuronal polarity via the PI3-kinase/Akt/GSK-3beta/CRMP-2 pathway. Biochem Biophys Res Commun 340: 62–68. 16343426 10.1016/j.bbrc.2005.11.147

[11] Toettcher J, Voigt C, Weiner O, Lim W (2010) The promise of optogenetics in cell biology: interrogating molecular circuits in space and time. Nat Methods 8: 35–38. 21191370 10.1038/nmeth.f.326 PMC3024327

[12] Ruthel G, Banker G (1998) Actin-dependent anterograde movement of growth-cone-like structures along growing hippocampal axons: a novel form of axonal transport? Cell Motil Cytoskeleton 40: 160–173. 9634213 10.1002/(SICI)1097-0169(1998)40:2<160::AID-CM5>3.0.CO;2-J

[13] Kennedy M, Hughes R, Peteya L (2010) Rapid blue-light-mediated induction of protein interactions in living cells. Nat Methods 7: 12–16. 10.1038/nmeth.1524 PMC3059133 21037589

[14] Inoue T, Meyer T (2008) Synthetic activation of endogenous PI3K and Rac identifies an AND-gate switch for cell polarization and migration. PLoS ONE 3: e3068. 18728784 10.1371/journal.pone.0003068 PMC2518103

[15] Watton SJ, Downward J (1999) Akt/PKB localisation and 3′ phosphoinositide generation at sites of epithelial cell-matrix and cell-cell interaction. Curr Biol 9: 433–436. 10226029 10.1016/s0960-9822(99)80192-4

[16] Donnelly ML, Hughes LE, Luke G, Mendoza H, Ten Dam E, et al (2001) The “cleavage” activities of foot-and-mouth disease virus 2A site-directed mutants and naturally occurring “2A-like” sequences. J Gen Virol 82: 1027–1041. 11297677 10.1099/0022-1317-82-5-1027

[17] Toettcher JE, Gong D, Lim W a, Weiner OD (2011) Light-based feedback for controlling intracellular signaling dynamics. Nat Methods 8: 837–839. 21909100 10.1038/nmeth.1700 PMC3184382

[18] Idevall-Hagren O, Dickson EJ, Hille B, Toomre DK, De Camilli P (2012) Optogenetic control of phosphoinositide metabolism. Proc Natl Acad Sci U S A 109: E2316–E2323. 22847441 10.1073/pnas.1211305109 PMC3435206

[19] Craig AM, Banker G (1994) Neuronal Polarity. Annu Rev Neurosci 17: 267–310 Available: http://www.annualreviews.org/doi/abs/10.1146/annurev.ne.17.030194.001411?journalCode=neuro. 8210176 10.1146/annurev.ne.17.030194.001411

[20] Nakata T, Hirokawa N (2003) Microtubules provide directional cues for polarized axonal transport through interaction with kinesin motor head. J Cell Biol 162: 1045–1055. 12975348 10.1083/jcb.200302175 PMC2172855

[21] Jacobson C, Schnapp B, Banker G a (2006) A change in the selective translocation of the Kinesin-1 motor domain marks the initial specification of the axon. Neuron 49: 797–804. 16543128 10.1016/j.neuron.2006.02.005

[22] Nakata T, Niwa S, Okada Y, Perez F, Hirokawa N (2011) Preferential binding of a kinesin-1 motor to GTP-tubulin-rich microtubules underlies polarized vesicle transport. J Cell Biol 194: 245–255. 21768290 10.1083/jcb.201104034 PMC3144414

[23] Arimura N, Kaibuchi K (2007) Neuronal polarity: from extracellular signals to intracellular mechanisms. Nat Rev Neurosci 8: 194–205. 17311006 10.1038/nrn2056

[24] Riedl J, Crevenna A, Kessenbrock K (2008) Lifeact: a versatile marker to visualize F-actin. Nat Methods 5: 605–607. 18536722 10.1038/nmeth.1220 PMC2814344

[25] Bridgman PC, Dailey ME (1989) The organization of myosin and actin in rapid frozen nerve growth cones. J Cell Biol 108: 95–109. 2642912 10.1083/jcb.108.1.95 PMC2115362

[26] Forscher P, Smith SJ (1988) Actions of cytochalasins on the organization of actin filaments and microtubules in a neuronal growth cone. J Cell Biol 107: 1505–1516. 3170637 10.1083/jcb.107.4.1505 PMC2115246

[27] Bodin S, Giuriato S, Ragab J, Humbel BM, Viala C, et al (2001) Production of phosphatidylinositol 3,4,5-trisphosphate and phosphatidic acid in platelet rafts: evidence for a critical role of cholesterol-enriched domains in human platelet activation. Biochemistry 40: 15290–15299. 11735411 10.1021/bi0109313

[28] Horiguchi K, Hanada T, Fukui Y, Chishti AH (2006) Transport of PIP3 by GAKIN, a kinesin-3 family protein, regulates neuronal cell polarity. J Cell Biol 174: 425–436. 16864656 10.1083/jcb.200604031 PMC2064238

[29] Hall A (1998) Rho GTPases and the actin cytoskeleton. Science 279: 509–514. 9438836 10.1126/science.279.5350.509

[30] Wu YI, Frey D, Lungu OI, Jaehrig A, Schlichting I, et al (2009) A genetically encoded photoactivatable Rac controls the motility of living cells. Nature 461: 104–108. 19693014 10.1038/nature08241 PMC2766670

[31] Doherty GJ, McMahon HT (2009) Mechanisms of endocytosis. Annu Rev Biochem 78: 857–902. 19317650 10.1146/annurev.biochem.78.081307.110540

[32] Macia E, Ehrlich M, Massol R, Boucrot E, Brunner C, et al (2006) Dynasore, a cell-permeable inhibitor of dynamin. Dev Cell 10: 839–850. 16740485 10.1016/j.devcel.2006.04.002

[33] Cupers P, Veithen A, Kiss A, Baudhuin P, Courtoy PJ (1994) Clathrin polymerization is not required for bulk-phase endocytosis in rat fetal fibroblasts. J Cell Biol 127: 725–735. 7962055 10.1083/jcb.127.3.725 PMC2120224

[34] Larkin JM, Brown MS, Goldstein JL, Anderson RG (1983) Depletion of intracellular potassium arrests coated pit formation and receptor-mediated endocytosis in fibroblasts. Cell 33: 273–285. 6147196 10.1016/0092-8674(83)90356-2

[35] Rejman J, Bragonzi A, Conese M (2005) Role of clathrin- and caveolae-mediated endocytosis in gene transfer mediated by lipo- and polyplexes. Mol Ther 12: 468–474. 15963763 10.1016/j.ymthe.2005.03.038

[36] Hutagalung AH, Novick PJ (2011) Role of Rab GTPases in membrane traffic and cell physiology. Physiol Rev 91: 119–149. 21248164 10.1152/physrev.00059.2009 PMC3710122

[37] Dittman J, Ryan T a (2009) Molecular circuitry of endocytosis at nerve terminals. Annu Rev Cell Dev Biol 25: 133–160. 19575674 10.1146/annurev.cellbio.042308.113302

[38] Patterson GH, Lippincott-Schwartz J (2002) A photoactivatable GFP for selective photolabeling of proteins and cells. Science 297: 1873–1877. 12228718 10.1126/science.1074952

[39] Laurino L, Wang XX, De la Houssaye B a, Sosa L, Dupraz S, et al (2005) PI3K activation by IGF-1 is essential for the regulation of membrane expansion at the nerve growth cone. J Cell Sci 118: 3653–3662. 16046480 10.1242/jcs.02490

[40] Amato S, Liu X, Zheng B, Cantley L, Rakic P, et al (2011) AMP-activated protein kinase regulates neuronal polarization by interfering with PI 3-kinase localization. Science 332: 247–251. 21436401 10.1126/science.1201678 PMC3325765

[41] Flynn KC, Pak CW, Shaw AE, Bradke F, Bamburg JR (2009) Growth cone-like waves transport actin and promote axonogenesis and neurite branching. Dev Neurobiol 69: 761–779. 19513994 10.1002/dneu.20734 PMC2845293

[42] Ruthel G, Banker G (1999) Role of moving growth cone-like “wave” structures in the outgrowth of cultured hippocampal axons and dendrites. J Neurobiol 39: 97–106. 10213456 10.1002/(sici)1097-4695(199904)39:1<97::aid-neu8>3.0.co;2-z

[43] Karunarathne WKA, Giri L, Kalyanaraman V, Gautam N (2013) Optically triggering spatiotemporally confined GPCR activity in a cell and programming neurite initiation and extension. Proc Natl Acad Sci U S A. 2013 110: E1565–74. 10.1073/pnas.1220697110 PMC3637763 23479634

[44] Turu G, Hunyady L (2010) Signal transduction of the CB1 cannabinoid receptor. J Mol Endocrinol 44: 75–85. 19620237 10.1677/JME-08-0190

[45] Sato M, Ueda Y, Takagi T, Umezawa Y (2003) Production of PtdInsP3 at endomembranes is triggered by receptor endocytosis. Nat Cell Biol 5: 1016–1022. 14528311 10.1038/ncb1054

[46] Zufferey R, Donello JJE, Trono D, Hope TJTTJ (1999) Woodchuck hepatitis virus posttranscriptional regulatory element enhances expression of transgenes delivered by retroviral vectors. J Virol 73: 2886–2892. 10074136 10.1128/jvi.73.4.2886-2892.1999 PMC104046

[47] De Felipe P, Ryan MD (2004) Targeting of proteins derived from self-processing polyproteins containing multiple signal sequences. Traffic 5: 616–626. 15260831 10.1111/j.1398-9219.2004.00205.x

[48] Kaech S, Banker G (2006) Culturing hippocampal neurons. Nat Protoc 1: 2406–2415. 17406484 10.1038/nprot.2006.356

[49] Nakata T, Terada S, Hirokawa N (1998) Visualization of the dynamics of synaptic vesicle and plasma membrane proteins in living axons. J Cell Biol 140: 659–674. 9456325 10.1083/jcb.140.3.659 PMC2140163

[50] Betz WJ, Mao F, Bewick GS (1992) Activity-dependent fluorescent staining and destaining of living vertebrate motor nerve terminals. J Neurosci 12: 363–375. 1371312 10.1523/JNEUROSCI.12-02-00363.1992 PMC6575621

[51] Hodgson L, Shen F, Hahn K (2010) Biosensors for characterizing the dynamics of rho family GTPases in living cells. Current protocols in cell biology Chapter 14: Unit 14.11.1–26. 10.1002/0471143030.cb1411s46 PMC2998069 20235099

