# Supplementary figures and images for: Optogenetic Control of PIP3: PIP3 Is Sufficient to Induce the Actin-Based Active Part of Growth Cones and Is Regulated via Endocytosis
Source: PLoS One. 2013 Aug 7;8(8):e70861. doi: 10.1371/journal.pone.0070861 (PMC3737352; doi:10.1371/journal.pone.0070861)

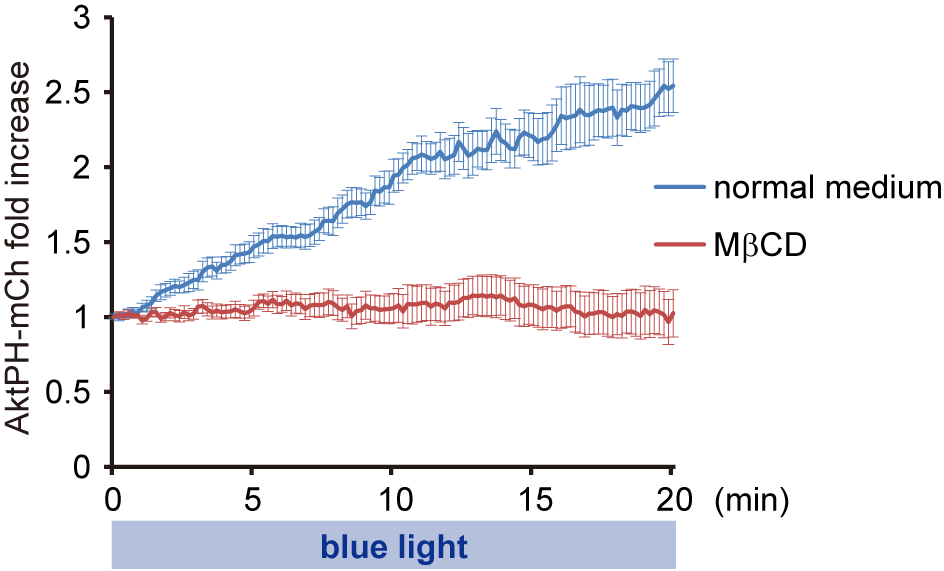

Supplement: Figure S1 — Choresterol-enriched membrane domains are necessary for local accumulation of PIP3. Time-course of fold changes in AktPH-mCh fluorescence in photoactivated growth cones expressing plasmid #3. Growth cones expressing plasmid #3 were photoactivated for 20 min with 5 mM MβCD (n = 11) or in normal imaging medium (n = 11, the same growth cone as the leftmost data in Fig. 5B). Data are expressed as means ± s.e.m. (TIF) [file pone.0070861.s001.tif]

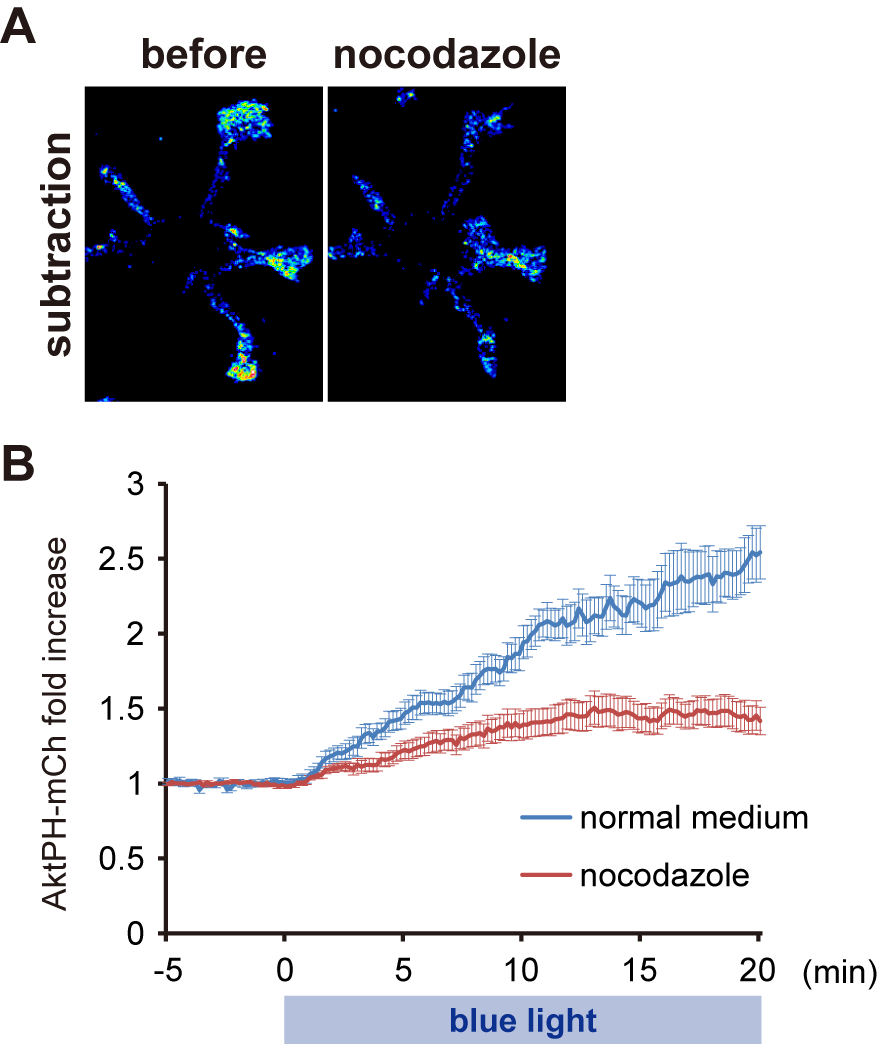

Supplement: Figure S2 — Microtubules-based transport is partially involved in the production of PIP3 at growth cones. (A) Subtracted AktPH images of a growth cone before and after nocodazle (3.3 µM) application in the absence of the photoswitch. (B) Time-course of fold changes in AktPH-mCh fluorescence in photoactivated growth cones expressing plasmid #3. Growth cones expressing plasmid #3 were photoactivated for 20 min with 3.3 µM nocodazole (n = 18) or in normal imaging medium (n = 11, the same growth cone as the leftmost data in Fig. 5B). Data are expressed as means ± s.e.m. (TIF) [file pone.0070861.s002.tif]
